# Predicted shifts in bacterial and algal contributions to DMSP and DMS dynamics during a coastal spring–summer bloom

**DOI:** 10.1093/ismejo/wrag141

**Published:** 2026-06-11

**Authors:** Xiao-Yu Zhu, Frances E Hopkins, Ruth Airs, Claire E Widdicombe, Bethany Wilkinson, Glen A Tarran, E Malcolm S Woodward, Ornella Carrión, Andrew R J Curson, Qianyao Ma, Libby Hanwell, Gui-Peng Yang, Joseph A Christie-Oleza, David J Lea-Smith, Xiao-Hua Zhang, Jonathan D Todd

**Affiliations:** School of Biological Sciences, University of East Anglia, Norwich Research Park, Norwich NR4 7TJ, United Kingdom; Quadram Institute Bioscience, Rosalind Franklin Road, Norwich Research Park, Norwich NR4 7UQ, United Kingdom; Centre for Microbial Interactions, Norwich Research Park, Norwich NR4 7UG, United Kingdom; Plymouth Marine Laboratory, Plymouth PL1 3DH, United Kingdom; Plymouth Marine Laboratory, Plymouth PL1 3DH, United Kingdom; Plymouth Marine Laboratory, Plymouth PL1 3DH, United Kingdom; Plymouth Marine Laboratory, Plymouth PL1 3DH, United Kingdom; Plymouth Marine Laboratory, Plymouth PL1 3DH, United Kingdom; Plymouth Marine Laboratory, Plymouth PL1 3DH, United Kingdom; School of Biological Sciences, University of East Anglia, Norwich Research Park, Norwich NR4 7TJ, United Kingdom; Quadram Institute Bioscience, Rosalind Franklin Road, Norwich Research Park, Norwich NR4 7UQ, United Kingdom; Centre for Microbial Interactions, Norwich Research Park, Norwich NR4 7UG, United Kingdom; School of Biological Sciences, University of East Anglia, Norwich Research Park, Norwich NR4 7TJ, United Kingdom; Centre for Microbial Interactions, Norwich Research Park, Norwich NR4 7UG, United Kingdom; Frontiers Science Center for Deep Ocean Multispheres and Earth System, and College of Marine Life Sciences, Ocean University of China, Qingdao 266100, China; School of Biological Sciences, University of East Anglia, Norwich Research Park, Norwich NR4 7TJ, United Kingdom; Centre for Microbial Interactions, Norwich Research Park, Norwich NR4 7UG, United Kingdom; Frontiers Science Center for Deep Ocean Multispheres and Earth System, and College of Marine Life Sciences, Ocean University of China, Qingdao 266100, China; Department of Biology, University of the Balearic Islands, Palma 07122, Spain; School of Biological Sciences, University of East Anglia, Norwich Research Park, Norwich NR4 7TJ, United Kingdom; Centre for Microbial Interactions, Norwich Research Park, Norwich NR4 7UG, United Kingdom; Frontiers Science Center for Deep Ocean Multispheres and Earth System, and College of Marine Life Sciences, Ocean University of China, Qingdao 266100, China; School of Biological Sciences, University of East Anglia, Norwich Research Park, Norwich NR4 7TJ, United Kingdom; Quadram Institute Bioscience, Rosalind Franklin Road, Norwich Research Park, Norwich NR4 7UQ, United Kingdom; Centre for Microbial Interactions, Norwich Research Park, Norwich NR4 7UG, United Kingdom; Frontiers Science Center for Deep Ocean Multispheres and Earth System, and College of Marine Life Sciences, Ocean University of China, Qingdao 266100, China

**Keywords:** DMSP synthesis genes, DMSP-producing microalgae and bacteria, coastal DMSP cycling, microalgal blooms

## Abstract

Ubiquitous marine microalgae and bacteria produce the abundant organosulfur compound dimethylsulfoniopropionate (DMSP) and/or catabolize it to climate-active gases, such as dimethylsulfide (DMS), with major consequences for global biogeochemistry and climate. However, their relative and dynamic roles in DMSP synthesis and catabolism remain poorly resolved, particularly during natural bloom events. Here, we combined metagenomics and metatranscriptomics, with measurements of intracellular/particulate DMSP (DMSPp), DMS concentrations, and DMSPp production rates, as well as microscopy and flow cytometry, to predict the key microbes and enzymes driving DMSP/DMS dynamics during a spring–summer bloom in the Western English Channel. Microalgae and bacteria expressing the DMSP synthesis genes *DSYB/DSYE* and *dsyB* were likely major and significant DMSP producers, respectively, except during the largest observed DMSP spike. This spike coincided with elevated *Synechococcus* and autotrophic flagellate biomass but minimal DMSP synthesis gene expression. Axenic *Synechococcus* strains contained no detectable DMSP, implying that flagellates with novel DMSP synthesis genes were likely responsible. Microbial DMSP import potential far exceeded catabolism, suggesting strong selection for DMSP uptake. Bacteria were the major predicted DMSP degraders, with DMSP demethylation potential dwarfing cleavage. However, the highest DMS concentrations were linked to *Haptophyta* expressing the DMSP lyase gene *Alma*, implying the significance of algal DMSP cleavage. Methanethiol-dependent DMS production was also likely important, with bacterial *mddH* transcripts coinciding with another major DMS spike. Overall, these results imply dynamic and contrasting roles of microalgae and bacteria, and their pathways, in coastal DMSP/DMS and sulfur cycling.

## Introduction

**Figure 1:**
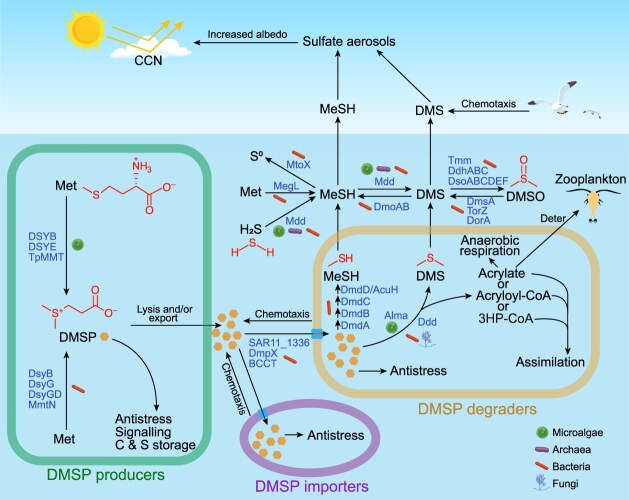
DMSP synthesis, import, and catabolism and their environmental importance in surface oceans. Microalgae and bacteria synthesize DMSP as an antistress, storage, or signalling molecule. Once released, DMSP can be imported by marine microbes through BCCT and/or ABC (SAR11_1336 and DmpX) family transporters for its antistress properties and/or as a carbon and sulfur source. DMSP is degraded by diverse marine microbes through cleavage (via Ddd or Alma enzymes) or demethylation (initially via DmdA) pathways, yielding the climate-active gases DMS and MeSH, respectively. DMS is also a potent chemoattractant for marine organisms and the major biogenic sulfur source transferred from the sea to air. DMSP cleavage can also yield acrylate, which can be used in anaerobic respiration or predator deterrence. H_2_S, MeSH, and DMSO are also DMS precursors. Conversely, DMS can be oxidized to DMSO or degraded to MeSH. MeSH can be degraded to sulfane sulfur (S^0^). Key enzymes in each reaction are shown in blue font. CCN, cloud condensation nuclei.

Billions of tonnes of the sulfonium zwitterion dimethylsulfoniopropionate (DMSP) are produced annually in Earth’s surface oceans [1, 2] (Fig. 1). Organisms produce DMSP for its antistress functions (e.g. for osmoprotection and antioxidation), sulfur and carbon storage, and signalling [1]. Key DMSP synthesis genes have been identified in microalgae (*DSYB, DSYE*, and *TpMMT*) [3–5], some cyanobacteria (*dsyG* and *dsyGD*) [4], and heterotrophic bacteria (*dsyB, dsyGD*, and *mmtN*) [4, 6, 7]. Microalgae are widely recognized as the major DMSP producers in Earth’s surface oceans, but intracellular/particulate DMSP (DMSPp) levels are highly variable in different taxa [8, 9]. Generally, *Haptophyta* (haptophytes) and *Dinophyta* (dinoflagellates) are high DMSP accumulators (HiDA, ≥50 mM) and contain *DSYB*, whereas *Bacillariophyta* (diatoms) are low DMSP accumulators (LoDA, <50 mM) and can contain *TpMMT* [9]. *DSYE* is found in both HiDA and LoDA algae, whilst all DMSP-producing bacteria are considered LoDA [4, 9]. DMSP-producing bacteria are likely more prominent contributors to the significant DMSP levels in marine sediments and aphotic and deep ocean environments [7, 10–12].

DMSP can be released into the environment by cell lysis and/or export (Fig. 1). Diverse organisms, including heterotrophic bacteria, phytoplankton, and macroalgae, can actively import DMSP and often concentrate it to millimolar cellular levels [13–18] for its antistress properties or for catabolism [1, 19] (Fig. 1). Known DMSP transporters are from BCCT [20] and ABC (SAR11_1336 [21] and DmpX [22]) transporter families. DMSP can be catabolized via demethylation and/or cleavage pathways by DMSP-producing and non-producing organisms [19], processes that are generally thought to be largely driven by bacteria [19, 23, 24]. Bacteria and microalgae can also oxidize DMSP to dimethylsulfoxonium propionate (DMSOP), which is thought to have a role in oxidative stress protection, but the biosynthesis enzymes are unknown [25].

Bacterial DMSP demethylation, initiated by DMSP demethylase [DmdA, converting DMSP to methylmercaptopropionate (MMPA)] and subsequently DmdB, DmdC, and DmdD/AcuH [26, 27], is predicted to be the dominant marine catabolic pathway [28–30], utilized for carbon and/or sulfur assimilation and can yield the climate-cooling gas methanethiol (MeSH) [26, 27] (Fig. 1). DmdA was initially identified in Roseobacter and SAR11 bacteria [27, 30]; however, functional DmdA homologues (DmdA_like) have recently also been detected in *Vibrio, Psychrobacter*, and SAR92 [31, 32]. MeSH can also be generated from the demethylation of methionine (Met) through L-Met gamma-lyase (MegL) [33, 34] (Fig. 1). DMSP cleavage yields the climate-cooling gas dimethyl sulfide (DMS) plus acrylate (via Alma-family enzymes [35], DddK [16], DddL [36], DddP [37], DddQ [38], DddU [39], DddW [40], and DddY [41]), 3-hydroxypropionate (3-HP)-CoA (via DddD [42]), or acryloyl-CoA (via DddX [43]) through nine ‘Ddd’ DMSP lyases in bacteria and fungi or Alma-family enzymes in microalgae (Fig. 1). Most DMS generated by marine microorganisms is released into the environment where it is a signalling molecule, nutrient for methylotrophs, or transferred to the atmosphere to influence cloud formation and global sulfur cycling [19, 44] (Fig. 1). The 3-carbon co-products of DMSP cleavage can be assimilated or used to deter predators [45] (Fig. 1). Hydrogen sulfide (H_2_S), MeSH, and dimethyl sulfoxide (DMSO) are also DMS biosources through bacterial, archaeal and/or algal *S*-methylation (via MddA [46, 47], MddH [48], MddM1 [49], and MddM2 [49]) or reduction (via DmsA [50, 51], DorA [52], and TorZ [53]) (Fig. 1). Conversely, DMS can be oxidized to DMSO by the multicomponent monooxygenase DsoABCDEF [54], DMS dehydrogenase DdhABC [55], and flavin-containing trimethylamine monooxygenase Tmm [56], or be degraded to MeSH by the DMS monooxygenase DmoAB [57] (Fig. 1). MeSH can be degraded to sulfane sulfur (S^0^) by the bacterial MeSH oxidase MtoX [58] (Fig. 1).

Many molecular ecological studies predict key microbial DMSP producers and degraders in seawater [12, 30, 59–62] and sediment [7, 10, 11, 63], but they have notable limitations. Most lack accompanying process measurements to validate their predictions. Polymerase chain reaction (PCR)-based studies are inherently biased by the primer specificity and typically exclude eukaryotic genes due to the absence of appropriate primers. In contrast, marine metagenomic and metatranscriptomic analyses are mostly performed on size-fractionated (e.g. 0.2–3 μm and >3 μm) samples, making it difficult to directly infer the contributions of larger celled algae and bacteria. Furthermore, the important microalgal DMSP synthesis gene *DSYE* [4] had not been discovered when many earlier DMSP cycling studies were conducted. With these limitations, there was a critical requirement to integrate multi-omics approaches with *in situ* process measurements to assess the potential contributions of microalgae and bacteria to DMSP production and cycling in marine environments.

In this study, surface coastal seawater was sampled from the L4 marine station (Fig. S1) in the Western English Channel (WEC) between March and July 2021, spanning reoccurring diatom (April–June) and dinoflagellate blooms (June–September) [64]. DMSPp and DMS concentrations, DMSPp production rate measurements, microscopy, flow cytometry, and multi-omics analyses on >0.2 μm samples were combined to predict the key organisms, enzymes, and pathways involved in DMSP synthesis, catabolism, and related processes at this established marine station (Tables S1 and S2).

## Materials and methods

### Seawater sample collection

Seawater samples from 10 m depth were collected weekly (conditions permitting) by 10 L Niskin bottles mounted on a rosette sampler that also housed a Seabird 19+ Conductivity–Temperature–Depth (CTD) profiler from March to July 2021 at the long-term time series station L4 in the WEC (Fig. S1, Table S1). Throughout the study, samples were consistently collected between 08:20 and 10:30 UTC, except on 21 June, when sampling occurred at 11:45 UTC.

### Measurements of nutrients, temperature, dissolved oxygen, salinity, Chlorophyll a, and plankton abundance and biomass

Nutrient samples were collected under clean conditions, kept cool and in the dark, and returned to the laboratory in Plymouth as soon as possible. Samples were filtered through 0.2 μm Millipore Fluoropore filters, and the filtrate was analysed by a 5-channel SEAL segmented flow analyser to determine the concentrations of ammonia, nitrate, nitrite, phosphate, and silicate. Temperature, dissolved oxygen, and salinity were directly measured by a Seabird 19+ CTD and its attached sensors during seawater collection. Chlorophyll a (Chl-a) concentrations were determined by filtering 100 ml of seawater through 25 mm GF/F filters, extracting pigments overnight in 90% acetone at 4°C, and measuring fluorescence with a Turner fluorometer as previously described [65].

*Synechococcus* and heterotrophic bacterial abundances were determined by flow cytometry (BD Accuri C6 flow cytometer) as previously described [66]. *Synechococcus* cells were identified by their natural pigment fluorescence and were gated based on their characteristic combination of low side scatter, orange phycoerythrin fluorescence, and red chlorophyll fluorescence, which distinguished them from heterotrophic bacteria and eukaryotic phytoplankton. Heterotrophic bacterial cells were enumerated separately after nucleic-acid staining with SYBR Green I and were distinguished from phototrophic cells based on their green fluorescence and side-scatter signatures. Cell concentrations were calculated from the analysed sample volume. Biomass was then estimated by multiplying cell concentrations by the mean cellular biomass (*Synechococcus*: 0.59 pg C cell^−1^; heterotrophic bacteria: 0.019 pg C cell^−1^) as previously described [64].

To determine the abundance of different microalgal taxa, seawater samples were fixed with acid Lugol’s iodine (all taxa except coccolithophores) or neutral formaldehyde (for coccolithophores) and analysed by light microscopy using the Utermohl counting technique [67]. Taxa-specific cell biovolumes were converted to cellular biomass (pg C cell^−1^) using previously described equations [68]. Microalgal biomass was then estimated by multiplying cell abundance by the taxa-specific cellular biomass.

### DMSPp, DMS, and DMSPp production rate measurements

For the characterization of DMS concentrations, DMSPp concentrations, and DMSPp production rates, triplicate 10 L samples were collected in autoclaved and acid-washed 250 ml glass-stoppered bottles, filled ensuring no headspace, and kept in the dark to avoid photo-oxidation in a cooler during transportation to the laboratory for analysis. DMSPp and DMS concentrations were measured by gas chromatography (GC), following methods described before [69, 70]. The DMS samples were prepared by gently filtering 5 ml of seawater through a 25 mm GF/F (glass fibre) filter and transferring to a glass purge tower, avoiding any contact with air, and immediately analysing. DMS was extracted from the 5 ml sample by purging with helium at a flow rate of 60 ml min^−1^ for 5 min whilst cryogenically trapping the DMS in a 1/16” PTFE sample loop submerged in liquid nitrogen. After 5 min, the sample loop was submerged in boiling water to desorb the DMS on a flow of helium carrier gas onto the GC column. For DMSPp, 7–10 ml of seawater was gravity filtered onto a 0.7 μm (nominal pore size) Whatman GF/F (25 mm diameter) to collect cells. The filter was placed in an 8 ml glass vial with 7 ml of Milli-Q water, before alkaline hydrolysis with the addition of 1 ml of 10 M NaOH solution. The sample was immediately capped, sealed, and left to rest for complete hydrolysis and equilibrium (at least 12 h) prior to analysis. For analysis, 2 ml of the supernatant was pipetted from the vial directly into the glass purge tower, and purged and cryogenically trapped, before desorption onto the GC column, as outlined for DMS.

Determination of *de novo* DMSP synthesis, expressed as the specific DMSP synthesis rate (μDMSP), and gross DMSPp production rates was performed as outlined before [70]. For each rate measurement, 9 × 500 ml polycarbonate bottles were filled gently by siphoning water directly from a 20 L water container. Tracer amounts of NaH^13^CO_3_, equivalent to ~6% of *in situ* dissolved inorganic carbon (DIC), were added to each 500 ml bottle. PAR (Photosynthetically Active Radiation) is routinely measured at the L4 station, but on the dates of the study, the PAR sensor was not working, so coincident data are not available. However, data from other years at this site demonstrate that PAR at 10 m depth is typically between 50 and 200 μE m^−2^ s^−1^. Using this as a guide, we incubated the polycarbonate bottles for DMSP synthesis rates in a Sanyo Versatile Environmental Test Chamber, set to the temperature of the seawater at the time of collection. On sunny days, the light settings in the growth cabinet were set to 150 μE m^−2^ s^−1^, and on cloudy days, a level of 65 μE m^−2^ s^−1^ was used. Triplicate samples were taken at 0 h and then at two further time points over a 6-h period. At each time point, 250 ml was gravity-filtered in the dark sequentially through a 3 μm membrane filter and then a 0.7 μm membrane filter. Each filter was then gently folded and placed in a 20 ml serum vial with 10 ml of Milli-Q and one NaOH pellet for alkaline hydrolysis, and the vial was crimp-sealed. Samples were stored at −20°C until analysis by proton transfer reaction mass spectrometer (PTR-MS) [71]. Calculation of μDMSP from ^13^C incorporation into DMSPp, and conversion to gross DMSPp production rates, were performed as previously described [70]. These measurements represent DMSPp production rates (i.e. production retained in the cells) rather than total DMSP production in the system. Newly synthesized DMSP released into the dissolved pool through secretion, leakage, or grazing-related processes would not be captured by this approach. In addition, microorganisms within the surface mixed layer are naturally exposed to variable irradiance due to vertical mixing, which cannot be fully replicated under fixed light conditions in bottle incubations. Therefore, the measured DMSPp production rates should be interpreted as estimates obtained under controlled incubation conditions rather than exact *in situ* rates.

### Amplicon, metagenomic, metatranscriptomic sequencing

For microbial/microalgal community and metabolic analysis, 3 L of seawater was filtered in triplicate for DNA and RNA extraction at each sampling date immediately on board the RV Plymouth Quest. Filtration was performed using a peristaltic pump (Watson-Marlow) with 0.22 μm polyethersulfone Sterivex filter units (Millipore). The process was completed within 2–8 h of sampling, after which filters were immediately flash-frozen in liquid nitrogen, before being transferred to a −80°C freezer. We acknowledge that the filtration period may represent a limitation for metatranscriptomic analyses because of the rapid turnover of mRNA in natural microbial communities.

Twelve of the 18 sampling dates were selected for downstream multi-omics analysis. Each filter was halved for parallel DNA and RNA extraction using the PowerSoil DNA Isolation Kit (Qiagen) and the Direct-zol RNA Kit (Zymo Research), respectively. For 16S rRNA amplicon sequencing, the V4 region was amplified using the 515F and 806R primers [72, 73]. All amplicons, metagenomes (total DNA), and metatranscriptomes (total RNA) were submitted to Novogene (Beijing, China) for quality control, library construction, and high-throughput sequencing on a HiSeq X Ten System (Illumina). Samples that failed to meet quality control standards were excluded from further sequencing.

### Metagenomic and metatranscriptomic analyses

Raw metagenomic and metatranscriptomic data were primarily quality-controlled by Fastp v0.23.2 [74]. SortMeRNA v4.3.6 [75] was further used to remove rRNA reads in metatranscriptomes. Clean reads of metagenomes or metatranscriptomes were then co-assembled for each sampling date with MEGAHIT v1.0.2 [76]. Prodigal v2.6.3 [77] and FragGeneScan [78] were used to predict genes for metagenomic and metatranscriptomic assemblies, respectively. Then, predicted genes from metagenomes and metatranscriptomes were clustered at 95% identity using CD-HIT v4.8.1 [79] to obtain the nonredundant gene catalogue. Taxonomic assignment of prokaryotic genes was conducted by the ‘easy-taxonomy’ module in MMseq2 [80] with GTDB release 220 [81] as the reference database. Taxonomic assignment of eukaryotic genes was conducted by Eukulele v2.0.9 [82] with PhyloDB (https://github.com/allenlab/PhyloDB) as the reference database.

The relative abundance of each gene in metagenomes and each transcript in metatranscriptomes were both estimated by the ‘metabat’ method in CoverM v0.6.1 [83]. The average relative abundance of 10 single-copy marker genes [84] was used to normalize the gene relative abundance in metagenomes as described previously [29]. These 10 marker genes were retrieved using hmmsearch v3.4.2 [85] with an e-value threshold of 1e-10. In contrast, transcript relative abundance in metatranscriptomes was normalized by the total read count of each metatranscriptome.

### Analyses of community composition

Qiime2 platform [86] was used to analyse the 16S rRNA gene amplicon data. The ‘DADA2’ plugin in Qiime2 was employed for quality filtering, denoising, chimera removal, and generation of amplicon sequence variants (ASVs). The ‘classify-sklearn’ module in Qiime2 was used to assign taxonomy for each ASV based on the Silva v138.1 database [87]. Phyloflash v3.4.2 [88] was applied to determine the prokaryotic community from metagenomes based on 16S rRNA gene reads with the Silva v138.1 [87] as the reference database. For the microeukaryotic community, we retrieved the 18S rRNA gene reads from metagenomes by Bowtie2 [89] and assigned taxonomy for each read using IDTAXA [90]. Both the identification and taxonomic assignment of 18S reads used the PR2 v4.13 as the reference database [91]. We filtered out nonprotistan sequences, defined as those that were assigned to the *Metazoa, Embryophyta*, and *Fungi*, before downstream analyses. Taxonomic profiles based on metatranscriptomic reads were analysed using Kraken2 [92] with the NCBI nr database as the reference database. Metatranscriptomic reads belonging to *Chordata, Arthropoda, Streptophyta, Mollusca*, and *Virus* were excluded. Downstream comparison analyses of microalgal and bacterial community compositions were performed with Microeco v1.14.0 [93].

### Metagenome-assembled genome recovery

The co-assembly contigs of each sampling date were individually imported to Semibin2 [94] to recover bacterial and archaeal metagenome**-**assembled genomes (MAGs). Then, MAGs from every sample were dereplicated using dRep v2.3.2 [95]. Genome completeness and contamination were estimated by CheckM v1.0.12 [96]. Only MAGs with medium to high quality (completeness ≥70%, contamination ≤5%) were retained for downstream analysis. Taxonomic assignment of each genome was determined by the ‘classify_wf’ module of GTDB-Tk v1.7.0 [81]. The MAG tree was also inferred by GTDB-Tk v1.7.0 [81] using the ‘infer’ module and was then visualized in ChiPlot [97]. Gene prediction and annotation of each MAG was performed by Prokka v1.12 [98]. The relative abundance/expression of MAGs in metagenomes/metatranscriptomes were estimated using the ‘relative abundance’ method in CoverM v0.6.1 [83].

### Identification of DMSP/DMS/MeSH metabolism–related genes

Enzymes assigned with Kyoto Encyclopedia of Genes and Genomes (KEGG) Orthology IDs (i.e. MegL, DsyB, DSYE, DsyGD, MmtN, DddD, DddX, DddL, DddQ, DddP, DddW, DddY, DmdA, DmdB, DmdC, DmdD, AcuH, TorZ, DorA, DmsA, DdhA, Tmm, DmoA, MtoX, and MddA) were annotated by BlastKOALA [99]. Because DddL and DddQ, as well as TorZ and DorA, share identical KO IDs, they were grouped as DddL/Q and TorZ/DorA in subsequent analyses. BCCT transporters were annotated by DIAMOND BLASTP [100] against the Transport Classification DataBase (TCDB) using an e-value threshold of 1e-10 [101]. The remaining enzymes (i.e. DSYB, DsyG, TpMMT, DmpX, SAR11_1336, Alma, DddK, DddU, DmdA_like, DsoB, MddH, MddM1, and MddM2) were identified by DIAMOND BLASTP [100] against their ratified sequences. Only homologues with ≥40% amino acid identity, ≥70% subject coverage, and ≥ 70% query coverage relative to ratified sequences were retained. For DmdA_like, DddK, MddH, and TpMMT, a set of previously identified nonhomologues were included as negative reference sequences in the DIAMOND BLASTP analyses. The KO IDs and sequences of all these enzymes are available in our custom database (https://github.com/zhuxiaoyu123/DMSP-database).

For genes identified by DIAMOND BLASTP against the ratified sequences, we evaluated the sensitivity of the observed relative transcript abundance patterns to the sequence identity threshold by repeating the analysis using the threshold applied in the main analysis (40%), a more permissive threshold (30%), and a more stringent threshold (50%). Overall, the relative transcript abundance patterns were highly consistent across the three cutoffs (Fig. S2), indicating that the main conclusions were robust and not strongly influenced by the choice of sequence identity cutoff.

### Intracellular DMSP measurements of *Synechococcus* spp.

Axenic cultures of marine *Synechococcus* strains WH7803, WH7805, WH8102, and CC9311 were routinely grown in ASW medium [102] using optimal growth conditions previously described [103]. For each strain, 100 ml mid-exponential phase culture was harvested by gentle centrifugation (3000*g* for 15 min at 20°C) and resuspended in 100 ml of nitrogen-depleted ASW medium. Cultures were prestarved for nitrogen for 24 h under optimal conditions (22°C at a light intensity of 10 μE m^−2^ s^−1^ with orbital shaking at 140 rpm). Subsequently, 12 ml aliquots of each culture were transferred into six 50 ml vented-cap culture flasks (GRYNIA). To three flasks, 120 μl of 880 mM NaNO_3_ was added (nitrogen-replete condition), whereas the remaining three flasks received 120 μl of Milli-Q water (nitrogen-depleted condition). All cultures were then incubated under optimal conditions for 3 days, after which cell concentrations were measured by flow cytometry (Becton Dickinson FACS-Verse cytometer) as previously described [104], and 10 ml of each culture was pelleted by centrifugation (4000*g* for 10 min at 4°C) and immediately stored at −20°C for subsequent DMSP analysis.

Pelleted *Synechococcus* cells (between 1.5 × 10^9^ and 1.7 × 10^10^ cells per pellet) were resuspended in 200 μl 50 mM Tris–HCl, 200 mM NaCl buffer (pH 7.5), transferred to a 2 ml glass GC vial and 100 μl 10 M NaOH was added (for alkaline lysis of DMS from DMSP) before immediately crimping the vial. Vials were incubated at 22°C in the dark for >6 h, and then, headspace DMS was measured by GC as previously described [3]. DMSP standards were analysed in the same way, and DMS peak areas were used to determine DMSP concentrations in the *Synechococcus* cells. None of the *Synechococcus* samples gave DMSP above the detection limit of the instrument (0.015 nmol DMSP in 300 μl sample or 0.05 μM). Based on the cell numbers in the samples and assumed *Synechococcus* cell volume (3 μm^3^) [105], this would mean that any DMSP produced by these strains would be at estimated intracellular concentrations of <0.3–3.3 μM or 0.9–10 zmol per cell.

### Statistical analyses

Pairwise Spearman correlations were analysed and plotted using the LinkET (https://github.com/Hy4m/linkET) package in R. Spearman rank correlation is a nonparametric approach that does not assume normally distributed data or linear relationships and is less sensitive to outliers. *P*-values were adjusted for multiple testing using the Benjamini–Hochberg method to control the false discovery rate.

## Results

### Microbial community analysis of the L4 spring bloom 2021

**Figure 2:**
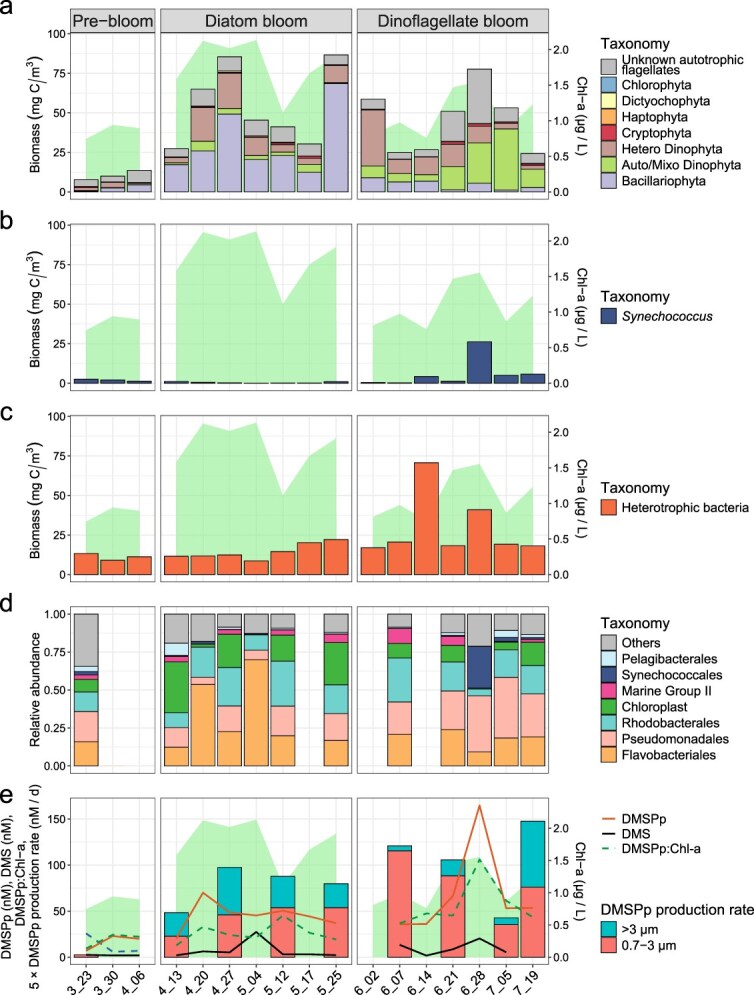
Variation in plankton communities, DMSPp, DMS, and Chl-a at station L4 from 23 March to 19 July 2021. (a) Biomass of microalgae determined by microscopy. (b) Biomass of *Synechococcus* determined by flow cytometry. (c) biomass of heterotrophic bacteria determined by flow cytometry. (d) Prokaryotic community composition determined by 16S rRNA gene amplicon sequencing. Some samples were not subjected to amplicon sequencing. (e) DMSPp and DMS concentrations and DMSPp:Chl-a ratios are shown by line plots. DMSPp production rates in small microalgae/free-living bacteria (0.7–3 μm) versus large microalgae/particle-associated bacteria (>3 μm) are presented in stacked bar plots. The DMSPp production rates shown are five times the measured values. The *x*-axis represents the sampling dates (e.g. ‘3_23’ indicates 23 March). The shaded areas represent the Chl-a concentrations throughout the sampling period.

The 2021 spring–summer bloom at station L4 consisted of a prebloom phase (23 March–6 April) with low microalgal biomass, a diatom (*Bacillariophyta*)-dominated phase (13 April–25 May), and a dinoflagellate (*Dinophyta*)-bloom phase (2 June–19 July) (Fig. 2a, Figs S3 and S4). A *Synechococcus* bloom was also observed on 28 June, with biomass comparable to that of autotrophic dinoflagellates (Fig. 2a and b). Heterotrophic bacteria showed increased biomass after the late diatom bloom, when diatoms had likely senesced and released intracellular organic matter (Fig. 2c). Community composition analyses (by 16S rRNA gene amplicon, metagenomic, and metatranscriptomic sequencing) largely supported these observations and revealed increased relative abundance of *Pseudomonadales, Flavobacterales*, and *Rhodobacterales* in the two bloom phases compared to the prebloom phase (Fig. 2d, Figs S5–S7).

### DMSPp and DMS concentrations in L4 samples

**Figure 3:**
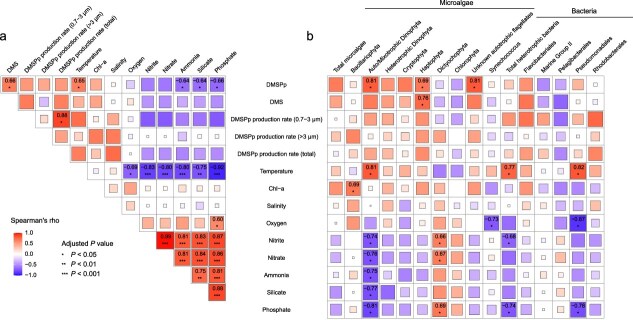
Spearman correlation analyses among environmental parameters (a) and between environmental parameters and the biomass of different plankton groups (b). Spearman’s rho values are shown only for significant correlations.

The prebloom phase showed the lowest observed DMSPp, DMS, and Chl-a concentrations and DMSPp production rates (Fig. 2e). The onset of the diatom bloom led to a rapid increase in DMSPp concentration, which was relatively stable throughout both bloom phases, except for a small spike during the diatom bloom (20 April, 70.4 nM) and a larger spike during the dinoflagellate bloom (28 June, 164.76 nM). Although total DMSPp production rates slightly increased during the dinoflagellate bloom compared to the diatom bloom phase, the contribution from small microalgae and free-living bacteria (0.7–3 μm) increased significantly and far exceeded that of larger microalgae and particle-associated bacteria (>3 μm) in the early and middle phases of the dinoflagellate bloom (Fig. 2e), suggesting that smaller microbes dominated DMSP production during this period. There was no significant correlation between DMSPp and Chl-a concentrations in the L4 samples (Fig. 3a), implying that the microalgal community were predominantly not HiDA and thus had likely low DMSPp:Chl-a ratios. This could not be corroborated by taxonomy because the dominant diatoms and dinoflagellate species (Fig. S4) had not previously been studied for DMSP production [9]. Furthermore, surface ocean Chl-a is only known as a good predictor of DMSP levels when HiDA microalgae are prominent [106–108]. In accordance with this, far higher DMSPp:Chl-a ratios (51.06 versus 25.09 on average) were observed during the dinoflagellate bloom compared to the diatom bloom samples (Fig. 2e), consistent with dinoflagellates and diatoms generally being HiDA and LoDA, respectively [9].

### Correlation analysis of environmental parameters, microbial taxa, and DMSPp/DMS levels

DMS concentrations (1.78–27.41 nM) positively correlated with DMSPp levels (Spearman’s rho = 0.66, *P* < .05), and were generally higher during the two bloom phases, but were always present at much lower levels than its likely DMSP precursor (20.51–164.76 nM) (Figs 2e and 3a). DMSPp levels showed a significant negative correlation with ammonia levels (Spearman’s rho = −0.64, *P* < .05, Fig. 3a), consistent with DMSP-producing algae and bacteria shifting metabolism to produce more nitrogen-independent osmolytes, such as DMSP, in response to nitrogen limitation [6, 9]. DMSP levels were also positively correlated with temperature and negatively correlated with silicate and phosphate (Fig. 3a). This pattern is likely explained by higher DMSPp concentrations associated with greater biomass of DMSP-producing phytoplankton at elevated temperatures, which, in turn, may contribute to the rapid drawdown of nutrients such as silicate and phosphate. Supporting this interpretation, all microalgal groups that were positively correlated with temperature were negatively correlated with these nutrients at L4 (Fig. 3b).

Correlations between DMSPp and various taxonomic groups were examined to explore potential associations between plankton groups and DMSP production (Fig. 3b). All significant correlations were observed in microalgal groups, supporting their well-established role in DMSP synthesis [9, 106]. Autotrophic flagellates (too small to be identified by microscopy) and auto-/mixotrophic *Dinophyta* displayed the strongest relationships with DMSPp (Spearman’s rho = 0.81, *P* < .05), followed by *Haptophyta* (Spearman’s rho = 0.69, *P* < .05) (Fig. 3b). Given that *Haptophyta* and *Dinophyta* are commonly considered as HiDA [9], these associations suggest that they may contribute to DMSP production at L4. No significant correlations were observed between DMSPp and other plankton groups. However, given the high biomass of certain groups (e.g. diatoms, *Synechococcus*, and heterotrophic bacteria; Fig. 2a–c) and/or the high DMSP synthesis potential of others (e.g. *Cryptophyta* and *Chlorophyta* [9]), their contributions to total DMSPp cannot be excluded.

Correlations between DMS and various taxonomic groups were examined to explore potential associations between plankton groups and DMS dynamics (Fig. 3b). Only *Haptophyta* showed a positive correlation with DMS (Spearman’s rho = 0.76, *P* < .05). No significant correlations were observed for other groups, possibly reflecting substantial variability in DMS production capacity and associated pathways among plankton taxa, as well as the fact that DMS concentrations represent the net balance between production and loss processes (e.g. air–sea exchange and photochemical or biological oxidation) [44]. Therefore, metagenomic and metatranscriptomic analyses of the presence, expression, and taxonomic affiliation of known DMSP/DMS metabolism-related genes may better predict the organisms and pathways influencing DMSP and DMS dynamics at L4.

### Microalgae and bacteria were respectively predicted as dominant or significant DMSP producers

**Figure 4:**
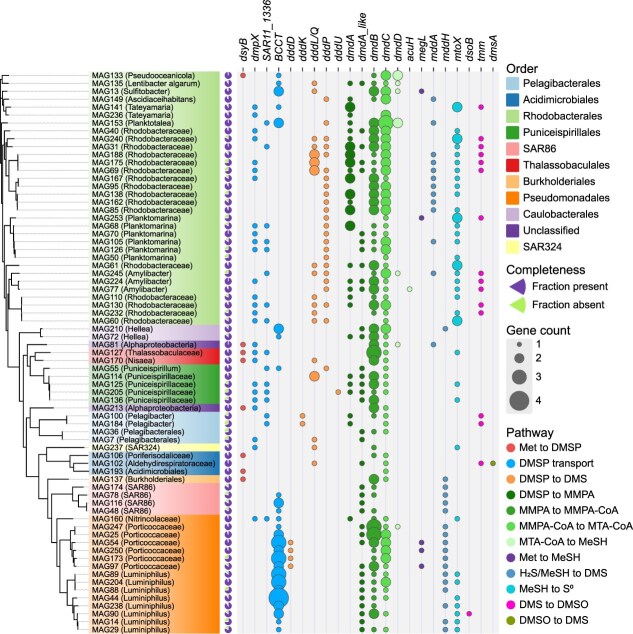
Maximum-likelihood tree of MAGs containing DMSP synthesis and catabolic genes. The lowest resolved taxonomic assignment for each MAG is shown in brackets. Genome completeness is indicated by pie charts. The copy numbers of DMSP synthesis and catabolic genes in each MAG are shown as bubble plots. Additional genes introduced in Fig. 1 were also examined in these MAGs to illustrate their co-occurrence.

**Figure 5:**
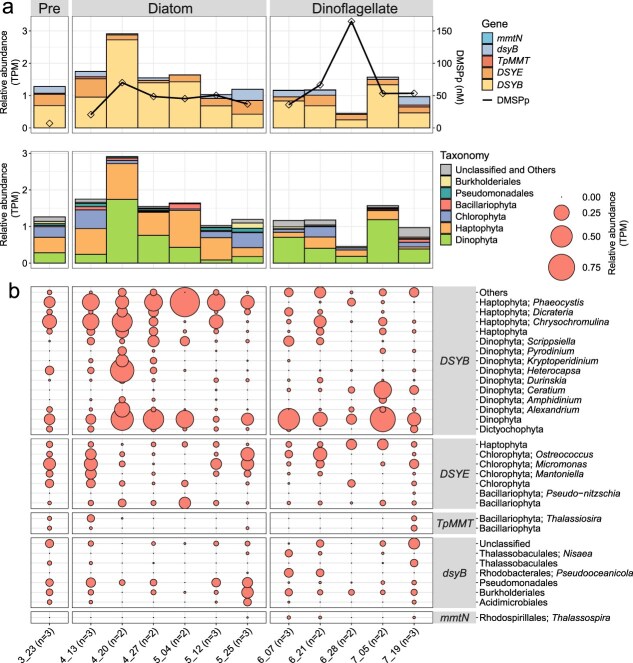
Relative transcript abundance of DMSP synthesis genes at station L4 in the WEC from 23 March to 19 July 2021. (a) Expression and order-level taxonomic profiles of all detected synthesis genes. DMSPp concentrations in corresponding samples are shown. (b) Taxonomic composition of each DMSP synthesis gene. Biological replicate counts (*n*) are shown in parentheses after each sample name. Pre, prebloom; Diatom, diatom bloom; Dinoflagellate, dinoflagellate bloom. TPM, transcripts per million reads.

**Figure 6:**
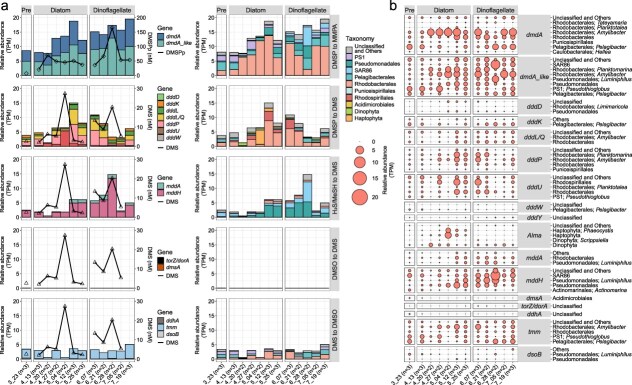
Relative transcript abundance of genes responsible for DMSP demethylation, DMSP/H_2_S/MeSH/DMSO-dependent DMS production, and DMS degradation at station L4 in the WEC from 23 March to 19 July 2021. (a) Expression and order-level taxonomic profiles of all detected genes. DMSPp or DMS concentrations in corresponding samples are shown. (b) Taxonomic composition of each gene. Biological replicate counts (*n*) are shown in parentheses after each sample name.

**Figure 7:**
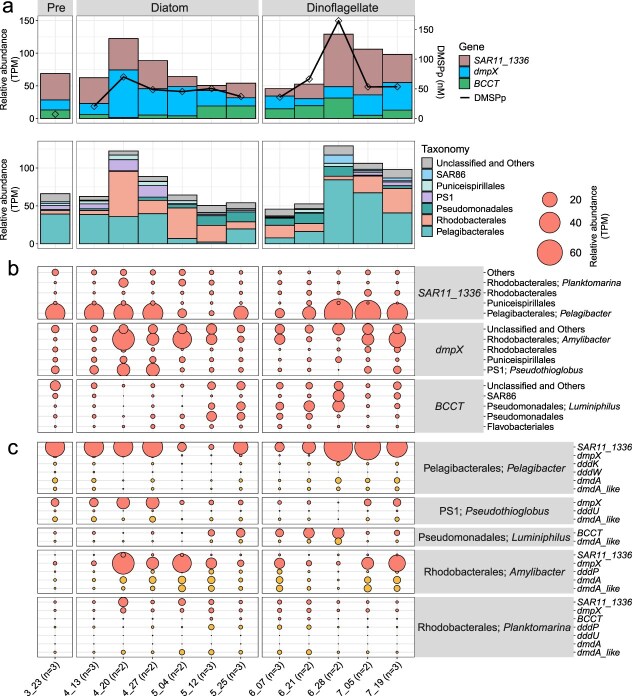
Relative transcript abundance of potential DMSP transporter genes at station L4 in the WEC from 23 March to 19 July 2021. (a) Expression and order-level taxonomic profiles of all detected DMSP transporter genes. DMSPp concentrations in corresponding samples are shown. (b) Taxonomic composition of each DMSP transporter gene. (c) Comparison of the expression levels of DMSP transporter (red) and catabolic (yellow) genes from the five predicted DMSP degraders at L4. Biological replicate counts (*n*) are shown in parentheses after each sample name.

Metagenomics and metatranscriptomics were performed on >0.22 μm fractionated samples to characterize the genetic potential and transcriptional profiles of bacteria and larger microalgae. Genes involved in DMSP/DMS/MeSH-related metabolism were identified and quantified in metagenomes (indicated as % of prokaryotes), metatranscriptomes (represented by transcripts per million reads ‘TPM’), and MAGs (Figs 4–7, Figs S8–S10, Tables S3–S7). Eukaryotic genes were sparsely detected in our metagenomic samples, likely because predicting eukaryotic genes from metagenomic assemblies remains challenging owing to their complex gene structures (e.g. introns and exons) and the prokaryote-focused bias of many gene-prediction tools [109]. They were therefore excluded from the metagenomic analyses.

Metagenomic analysis of samples from station L4, like that of the global surface ocean [4, 110], predicted that only a small proportion (0.53% of prokaryotes on average; 0.52% with *dsyB* and 0.01% with *mmtN*) of L4 prokaryotes could produce DMSP (Fig. S8, Table S3). Consistently, only 8 of 260 MAGs were predicted with *dsyB* and none had *mmtN* or *dsyG/GD* genes (Fig. 4, Table S7). *DsyB* genes detected in metagenomes were mainly affiliated with members of the *Pseudomonadota*, particularly *Pseudomonadales, Burkholderiales, Rhodobacterales*, and *Thalassobaculales*, with additional sequences assigned to *Acidimicrobiales* (*Actinobacteriota*) (Fig. S8), suggesting that the potential for DMSP production was distributed across diverse marine bacterial taxa.

Metatranscriptomics was used to estimate the transcript abundance of eukaryotic and prokaryotic DMSP synthase genes. Eukaryotic synthase gene transcripts were more abundant than their prokaryotic counterparts (1.25 versus 0.14 TPM). Although transcript abundance is not a direct measure of enzymatic activity or process rates, the higher abundance of eukaryotic DMSP synthase transcripts, together with the positive correlations between DMSPp concentrations and the biomass of several phytoplankton groups (Fig. 3b), and the high intracellular DMSP concentrations typically found in microalgae [9], suggest that microalgae were likely major DMSP producers at L4. *DSYB*, mainly from *Dinophyta* and *Haptophyta*, was the most highly expressed known DMSP synthesis gene across all L4 samples (0.98 TPM average, Fig. 5). Algal *DSYE* transcripts (0.24 TPM), largely from *Chlorophyta, Bacillariophyta*, and *Haptophyta*, were always much less prominent than for *DSYB* but generally more abundant than for bacterial *dsyB* (0.14 TPM) (Table S5). Algal *TpMMT* (0.01 TPM) and bacterial *mmtN* (0.001 TPM) transcripts were far less frequent, consistent with global surface ocean analysis [4, 111] (Fig. 5). Although microalgae accounted for the majority of DMSP synthesis transcripts, bacterial *dsyB* transcripts, mainly from *Burkholderiales* and *Pseudomonadales*, were consistently detected and, in some samples, reached levels comparable to algal *DSYE* (Fig. 5), suggesting that bacteria may represent significant contributors to DMSP synthesis. These data extend the potentially important role of bacteria in DMSP synthesis beyond previously reported aphotic and sediment environments [7, 12], showing that bacteria may also contribute significantly to DMSP synthesis in sunlit seawater alongside microalgae.

Cumulatively, DMSP synthase gene transcripts, particularly *DSYB*, were more abundant during the diatom bloom versus the dinoflagellate bloom (Fig. 5a). However, DMSPp concentrations remained broadly comparable between the two phases, except for a pronounced spike on 28 June. In addition, total DMSPp production rates were generally higher during the dinoflagellate bloom (Fig. 2e). Moreover, no significant correlations were observed between DMSP synthesis gene transcript abundance and DMSPp/DMS concentrations or DMSPp production rates (Fig. S11). These discrepancies could reflect shifts in the composition of DMSP-producing taxa between the two bloom phases, with *DSYB* transcripts from the haptophytes *Phaeocystis* and *Chrysochromulina*, and the dinoflagellate *Heterocapsa* being more abundant during the diatom-dominated phase (Fig. 5b). Conversely, *DSYB* transcripts from the dinoflagellate *Ceratium* were more abundant during the dinoflagellate bloom. This temporal succession of *DSYB*-expressing taxa may indicate a shift among *DSYB*-expressing taxa carrying isoforms with potentially different enzymatic properties, potentially decoupling *DSYB* transcript abundance from DMSPp concentrations and DMSPp production rates (Fig. 5a, Fig. S11). *DSYE* transcript abundance did not change significantly during the blooms, but diatom *DSYE* and *TpMMT* transcripts, although low (less than prokaryotic gene transcripts), were generally more prominent during the diatom bloom (Fig. 5a). Furthermore, all detected eukaryotic synthesis transcripts were not from the dominant predicted microalgal genera observed in the L4 samples (Fig. S4), implying that many of the algal taxa were either (i) not producing DMSP (i.e. not expressing their synthesis genes), (ii) utilizing unknown DMSP synthesis genes (e.g. from the decarboxylation pathway in *Crypthecodinium cohnii* [112]), or (iii) lacked the capacity for DMSP production. Cases (a) and (c) would highlight the potential contribution of subdominant taxa and potentially HiDA dinoflagellates, haptophytes and chlorophytes, and, to a lesser extent, LoDA bacteria, in DMSP production during the spring microalgal bloom at L4.

The highest DMSPp and DMSPp:Chl-a levels, observed in the 28 June samples, coincided with the lowest abundance of known DMSP synthesis gene transcripts. This peak aligned with a substantial increase in *Synechococcus* abundance (Fig. 2b), a predicted LoDA [9] previously estimated to accumulate 2%–21% of DMSP in the WEC [113]. *Synechococcus* spp. are known to import DMSP from the environment [17], but their ability to produce DMSP remains uncertain. Low intracellular DMSP levels (0.03–0.7 mM) have been reported in two nonaxenic cultures [114], whereas another study found no detectable DMSP in 11 nonaxenic *Synechococcus* samples [9]. To establish if *Synechococcus* can synthesize DMSP, we analysed axenic cultures of four species that were phylogenetically close to L4 *Synechococcus* (Fig. S12). These strains were grown in artificial seawater media under nitrogen replete or depleted conditions. Significantly, none of these cyanobacteria accumulated DMSP above the detection limit of the GC instrument, irrespective of nitrogen levels, which are known to regulate DMSP production in many producers [1] (see Materials and Methods). If DMSP was present in these cyanobacteria, it would, at best estimate, be equivalent to 0.3–3.3 μM or 0.9–10 zmol per cell, see Materials and Methods, indicating that these *Synechococcus* are unlikely to be major contributors to DMSP levels in the L4 samples. However, it is possible that the exact *Synechococcus* populations at station L4, which were not cultured here, possess unknown DMSP synthesis genes and accumulated higher DMSP levels than we observe in the closely related tested strains. The DMSPp spike on 28 June also coincided with a peak in abundance of autotrophic flagellates (Fig. 2a), which showed significant correlation with DMSPp (Spearman’s rho = 0.81, *P* < .05, Fig. 3b). Thus, rather than *Synechococcus*, it is more likely that some of these small microalgae present were HiDA with unknown DMSP synthesis genes, as is the case for the dinoflagellate *C. cohnii* that utilizes the decarboxylation pathway for DMSP synthesis [112], and were responsible for this larger DMSPp spike.

### Bacteria and microalgae were respectively predicted dominant or significant DMSP degraders

Bacterial *dmdA/dmdA_like* genes (37.3% of prokaryotes) and transcripts (13.6 TPM) were more abundant than *ddd* genes (22.6% of prokaryotes) and their transcripts (5.5 TPM) in L4 samples (Fig. 6a, Fig. S9a, Tables S3 and S5), consistent with previous reports in the global ocean [29, 30] and supporting the dominant role of bacterial demethylation in DMSP catabolism across diverse marine environments [28]. The two main lyase genes at L4 were *dddP* and *dddU*, with the latter previously shown not to be prominent in the global-surface open ocean [29], indicative of differences in bacterial community composition between open-ocean and coastal waters. Although fungi contributed significantly to ocean biomass (9/44 of bacterial biomass in open ocean) [115], their *dddP* [37] and *dddW* [116] genes were not detected in our metatranscriptomes, suggesting their minimal role in L4 DMSP cycling.

Bacteria were predicted as the dominant DMSP degraders based on far higher prokaryotic *dmdA/dmdA_like* (13.6 TPM) and *ddd* relative transcript abundances (5.5 TPM) compared to eukaryotic *Alma* (0.87 TPM) in all samples (Fig. 6a, Table S5). The *dmdA/dmdA_like* and *ddd* genes were largely from *Rhodobacterales* (mainly *Amylibacter* and *Planktomarina*) (Fig. 6a and b), whose biomass greatly increased during both blooms (Fig. 2c and d) and whose relative abundance was previously shown to strongly correlate with HiDA microalgae in coastal seawaters [117]. Among the 31 *ddd/dmdA*-containing *Rhodobacterales*, 67% (21) encoded both *ddd* and *dmdA/dmdA_like* genes, and 58% (18) harboured multiple *ddd* and/or *dmdA/dmdA_like* genes (Fig. 4). These proportions were markedly higher than those observed in the 33 *ddd/dmdA*-containing MAGs from other taxa, of which 27% (9) encoded both genes and 12% (4) contained multiple *ddd* and/or *dmdA/dmdA_like* genes. In addition, compared with these 33 MAGs from other taxa, the *ddd/dmdA*-containing *Rhodobacterales* were also enriched in the DMS oxidation gene *tmm* and the MeSH degradation gene *mtoX* (Fig. 4). Together, these results suggest that *Rhodobacterales* may play a relatively important role in DMSP cleavage and demethylation, as well as in downstream DMS and MeSH metabolism. Other *dmdA* or *ddd* genes were mainly from *Pseudomonadales, Pelagibacterales*, and PS1 or *Pelagibacterales* and *Rhodospirillales*, respectively (Fig. 6a and b).

Alma-family DMSP lyase gene transcripts, mainly from *Haptophyta* and *Dinophyta*, were always less abundant than for *ddd*, except on 20 April and 4 May, which corresponded with a minor and the largest observed DMS spikes in the diatom bloom, respectively (Fig. 6a). Furthermore, *Haptophyta* biomass was shown to have strong relationships with DMS (Spearman’s rho = 0.76, *P* < .05, Fig. 3b). The minor DMS peak was associated with *Dinophyta* and the largest with *Haptophyta*, predominantly *Phaeocystis* (Fig. 6a and b), a genus often associated with active DMSP cleavage [118, 119]. Bacterial *ddd* transcripts were barely detected in these two samples, suggesting that microalgae played a key role not only in DMSP synthesis but also in its cleavage. Moreover, *Dinophyta*-derived *Alma* genes were consistently expressed across the spring bloom (Fig. 6b). These findings support a previous study, which proposed that bacteria-mediated DMS production alone could not account for the high DMS concentrations seen in surface seawaters [107].

### MeSH may be an important precursor of DMS at L4

We did not see any corelation between DMS concentration and DMSP lyase transcript relative abundance (Fig. S11). This was likely due to variability in the enzymatic efficiencies of the different known DMSP lyases [120], the activity of as yet unidentified DMSP lyases, or of other biological DMS generating or catabolic pathways [44] (Fig. 1), in combination with multiple dynamic and variable nonbiological loss pathways for DMS from the surface ocean (e.g. ventilation to the atmosphere via the sea–air flux and photo-chemical reactions) [44]. Indeed, the second largest observed DMS spike corresponded to the 28 June sample, with the highest DMSP level, lowest DMSP synthesis potential (described above), and, significantly, one of the lowest observed levels of *ddd/Alma* transcripts. Thus, other known DMS production genes were examined in the L4 samples (Fig. 6a and b, Fig. S9a and b). The 28 June sample exhibited the highest abundance of *mdd* genes/transcripts, whose products convert H_2_S and MeSH to DMS [46–48] (Fig. 1). These *mddH* gene products, primarily from SAR86 and *Pseudomonadales*, may have contributed to the second highest observed DMS peak in the L4 samples. Excepting the 28 June samples, *mdd* genes and transcripts were generally less abundant than those for *ddd* genes (17.2% versus 22.6% of prokaryotes; 4.7 versus 5.5 TPM) but even these levels were clearly sizable and imply that they played a significant role in sulfur cycling at L4 (Fig. 6a, Fig. S9a, Tables S3 and S5). *mddH* genes/transcripts were far more abundant than *mddA* (14.4% versus 2.7% of prokaryotes; 4.0 versus 0.6 TPM), which is thought to be more prominent in terrestrial environments [46–48]. The genetic potential for MeSH production from DMSP demethylation and Met lysis, as well as MeSH degradation, is extensive in L4 samples (Figs 1 and 6a, Fig. S13, Tables S3 and S5). Given this prediction and that H_2_S concentrations are often below detection limits in well-oxygenated surface ocean samples [121, 122], MeSH was therefore likely the key Mdd substrate, suggesting significant potential for MeSH-dependent DMS production in this marine environment.

*DMSO* reductase genes and transcripts, which encode enzymes that reduce DMSO to DMS, were also seen in the L4 samples but at levels lower than for DMSP cleavage and H_2_S/MeSH-dependent DMS production pathways (0.1% of prokaryotes; 0.01 TPM) (Fig. 6a and b, Fig. S9a and b, Tables S3 and S5). This is consistent with a previous finding at L4, where >94% of DMSO was dissimilated to CO_2_ [123].

DMS levels are also influenced by its consumption, not measured here. Nevertheless, microbial DMS oxidation may play an important role in DMS removal in L4 samples, supported by the notable mean relative abundance (10.9% of prokaryotes) and relative expression (2.8 TPM) of *tmm* genes (Fig. 6a, Fig. S9a, Tables S3 and S5). Reduced *tmm* expression observed in the 20 April and 4 May samples may have further contributed to the elevated DMS concentrations on those dates (Fig. 6a).

### Bacterial DMSP import potential outweighs catabolism

The putative DMSP transporter genes *BCCT, SAR11_1336*, and *dmpX* were predicted in 44.1%, 8.0%, and 22.9% of prokaryotes, respectively. Collectively, these genes were more prevalent than known bacterial DMSP cleavage (22.6%), demethylation (37.3%), and synthesis (0.53%) genes (Fig. S9a, Table S3). Their combined transcript abundance (78.6 TPM) was also markedly higher than that of bacterial DMSP cleavage (5.5 TPM), demethylation (13.6 TPM), and synthesis (0.14 TPM) genes (Fig. 7a, Table S5). This pattern is unsurprising given some bacteria that do not catabolize DMSP can still use it for osmoprotection [124, 125]. Only DmpX is known to be highly specific for DMSP import, whereas BCCT and SAR11_1336 transporters can also import other compounds such as betaines [20, 21]. Therefore, the high abundance of these transporter genes may reflect a broader compatible solute uptake capacity in these bacteria rather than DMSP-specific import alone.

Although dissolved DMSP (DMSPd), rather than DMSPp, is the form directly available for transport, DMSPd and DMSPp often exhibit broadly similar distribution patterns in marine environments [60, 106]. In L4 samples, intracellular DMSPp concentrations broadly covaried with transcripts of putative DMSP transporter genes, primarily affiliated with predicted DMSP-degrading *Pelagibacterales, Rhodobacterales*, and *Pseudomonadales* (Fig. 7a), suggesting that DMSP availability may shape microbial osmolyte uptake dynamics. ABC transporters (*SAR11_1336* and *dmpX*) exhibited lower relative gene abundance but significantly higher transcript levels compared to *BCCT* (Fig. 7a, Fig. S10a). This may reflect greater transcriptional investment in the ABC transport systems by their host strains, potentially enabling them to compete more effectively for available DMSP. Consistently, *Pelagibacter, Pseudothioglobus*, and *Amylibacter*, three predicted DMSP degraders at L4 (Fig. 6), encoded fewer ABC-family DMSP transporter genes than DMSP catabolic genes (Fig. S10c) but showed substantially higher expression of ABC-family DMSP transporter genes than of DMSP catabolic genes (Fig. 7c). Although this pattern was not observed in two other predicted DMSP degraders, *Luminiphilus* and *Planktomarina*, the relative transcript abundances of their DMSP transporter genes were higher than those of their DMSP catabolic genes in most samples. These results further imply the importance of DMSP import and intracellular accumulation for catabolism and potentially as an antistress compound.

## Discussion

This study uniquely uses molecular approaches (metagenomics and metatranscriptomics) combined with direct measurements of *in situ* chemical and biological variables and process measurements to predict the key microalgae and bacteria involved in DMSP synthesis and catabolism in temperate coastal surface waters during a spring microalgae bloom.

Microalgae were inferred as the primary DMSP producers in the WEC coastal waters, due to their strong correlations to DMSPp concentration (Fig. 3b), and high expression of *DSYB*, particularly from HiDA dinoflagellates and haptophytes (Fig. 5). Even though diatoms (LoDA) were abundant, notably through the diatom bloom phase (Fig. 2a), their contribution to DMSP production was likely limited as indicated by relatively low expression of diatom *DSYE* and *TpMMT* genes (Fig. 5). These data were consistent with HiDA abundance/activity, rather than total microalgal abundance, determining DMSPp production rates in the surface ocean [9, 108]. Bacteria are considered as important DMSP producers in aphotic marine waters and sediments with negligible production from microalgae [7, 12, 106]. Unexpectedly, bacteria, particularly *Pseudomonadales* and *Burkholderiales* with *dsyB*, were likely significant contributors to total DMSP levels, with *dsyB* transcripts generally more abundant (~10% of total DMSP synthesis gene transcripts and 14.1% of eukaryotic *DSYB* transcripts) than algal *TpMMT*, and sometimes comparable to *DSYE* (Fig. 5, Table S5). Although the enzymatic efficiency of DsyB is slightly lower than DSYB [120], their substantial expression suggests a potentially important role for LoDA bacteria in DMSP production. Given the minuscule DMSP amounts axenic *Synechococcus* strains potentially accumulated, they were unlikely to be important DMSP producers at L4 despite their notable biomass (Fig. 2b) and previous reports of DMSP production [113, 126]. *Synechococcus* are much more likely significant importers and accumulators of DMSP [127] because they contain BCCT family transporters (Fig. S14), are not thought to catabolize DMSP, and lack all known primary DMSP catabolic enzymes.

In contrast to algal-driven DMSP synthesis, bacteria, particularly *Rhodobacterales, Rhodospirillales,* and *Pelagibacterales* with *dmdA/dmdA_like, dddP* and/or *dddU* genes, were predicted as the major DMSP degraders in L4 coastal waters (Fig. 6a), as previously suggested in the global ocean [28–30]. Consistent with previous studies [28–30], bacterial-driven DMSP demethylation potential was always far greater than DMSP cleavage potential and was relatively consistent between samples. DMSP cleavage was also predominantly predicted to be bacterial, with only 2 of 12 samples exhibiting higher cleavage potential in DMSP-producing microalgae, primarily *Haptophyta*, than bacteria (Fig. 6a). It is unclear why these microalgae in the 20 April and 4 May samples likely preferentially cleaved intracellular DMSP, generating cleavage products that may deter grazers [128, 129], attract heterotrophic microbes [130, 131], or attract micropredators and the predators of those micropredators [132]. Nevertheless, these two algal events displayed significant spikes in DMS levels, highlighting not only the importance of both microalgae and bacteria in DMSP cleavage but also the more dynamic nature of the former. In addition to DMSP, MeSH was also a likely alternative DMS precursor. This was supported by: (i) significant expression of *mdd* (largely *mddH*), which, in some cases (e.g. 28 June), exceeded those of DMSP lyase genes and coincided with a pronounced DMS spike (Fig. 6a); (ii) a high predicted potential for MeSH production via DMSP demethylation and Met cleavage (Fig. S13); and (iii) the widespread presence of MeSH in the global ocean [133, 134]. In contrast to Mdd pathways, DMSO was likely a less significant biological source of DMS, indicated by the low observed DMSO reductase expression levels (Fig. 6a) and previous observations [123].

The reason for consistently lower expression levels of DMSP synthesis genes compared to those for DMSP catabolism and import, observed across all L4 samples and in previous studies on other marine environments, remains unclear. One possibility is that transcript abundance may not accurately reflect enzyme abundance or activity. Additionally, DMSP synthesis is known to be tightly regulated by environmental conditions [9], and bacterial DMSP synthesis genes are typically restricted to a narrower range of taxa than for DMSP import and catabolism, which are widespread among marine bacteria [1, 6, 7, 19, 20, 22–24] and support key cellular functions such as growth, sulfur and carbon assimilation, and stress protection [19]. DMSP transporter genes were expressed at higher levels than those for catabolism in predicted DMSP degrading bacteria, including *Pelagibacter, Amylibacter, Luminiphilus, Pseudothioglobus*, and *Planktomarina* (Fig. 7c, Fig. S9c). This suggests a requirement to concentrate DMSP within the cell, likely for stress protection and subsequent catabolism, especially given the high *K_m_* values of the associated catabolic enzymes [120].

Although our multi-omics approach enabled detailed functional and taxonomic predictions of DMSP production, import, and cycling, we acknowledge several limitations. Transcript abundance does not directly reflect protein levels or enzymatic activity. Furthermore, there is still significant work required to establish the functional divergence within DMSP-related gene families. For example, TpMMT from the diatom *Thalassiosira pseudonana* is still the only characterized member of this protein family [5], and it is still difficult to predict DMSP lyase activity within the Alma family [35]. Moreover, we know that there are still important reporter genes for DMSP synthesis, transport, and catabolism to be identified (e.g. DMSP synthesis genes in DMSP-producing *Trichodesmium* cyanobacteria) [135] or dinoflagellates using the decarboxylation pathway [112], which incidentally may have been utilized by the flagellates likely responsible for the major 28 June DMSP spike at L4 (Fig. 2e). The unavoidable omissions of these genes in this analysis also led to an underestimation of certain pathways in the DMSP cycle. DMSP can be biologically transformed into various other DMSP-related compounds, such as DMSOP and gonyol, which generally occur in seawater at much lower concentrations than DMSP [25, 136] but may also contribute to DMSP cycling. These compounds were not included in this study due to limited knowledge on their key biosynthetic genes and our lack of reliable detection methods.

In summary, this study highlights the critical roles of both phototrophic microalgae and heterotrophic bacteria in DMSP synthesis and catabolism, revealing a dynamic and at times contrasting contribution of these groups that cannot be reliably predicted from taxonomy and environmental parameters alone. Microalgae generally dominated DMSP synthesis, but it remained difficult to predict when and why bacterial production became more significant. Although bacterial DMSP demethylation consistently dominated catabolism, DMSP cleavage was also important and mostly driven by bacteria. However, sporadic high algal lyase activity occurred unpredictably, even during algal blooms. Moreover, nonconventional pathways, such as MeSH-dependent DMS production, previously considered minor contributors to the marine DMS pool, showed relatively high transcriptional activity in our study. This suggests they may play a more significant role in DMS production than previously recognized, although their occurrence remained unpredictable. Given that these findings are based on transcriptomic data, further field investigations and *in situ* rate measurements are warranted to quantify the actual metabolic contributions of these pathways. Prediction of the microbes and pathways potentially driving the synthesis and cycling of major organosulfur compounds was enabled by integrating process monitoring with metatranscriptomics and comprehensive molecular marker analysis. Given the considerable variability in microbial sulfur cycling revealed here at a single coastal site during one spring–summer bloom, it is essential to conduct similar studies across diverse marine waters and sediments to develop a better understanding of marine microbial organosulfur cycling.

## Supplementary Material

Supplementary_material_wrag141

## Data Availability

Raw reads of metagenomes, 16S rRNA gene amplicons, and metatranscriptomes are available at NCBI under the project accessions PRJNA901156, PRJNA901154, and PRJNA901170, respectively. All source data used in this work are available at Zenodo (https://doi.org/10.5281/zenodo.19770266).
