# Supplementary material for: Predicted shifts in bacterial and algal contributions to DMSP and DMS dynamics during a coastal spring–summer bloom
Source: ISME J. 2026 Jun 11;20(1):wrag141. doi: 10.1093/ismejo/wrag141 (PMC13310141; doi:10.1093/ismejo/wrag141)
Supplement: Supplementary_material_wrag141 [file supplementary_material_wrag141.zip › L4_Supplementary_figures.docx]

**Supplementary figures**


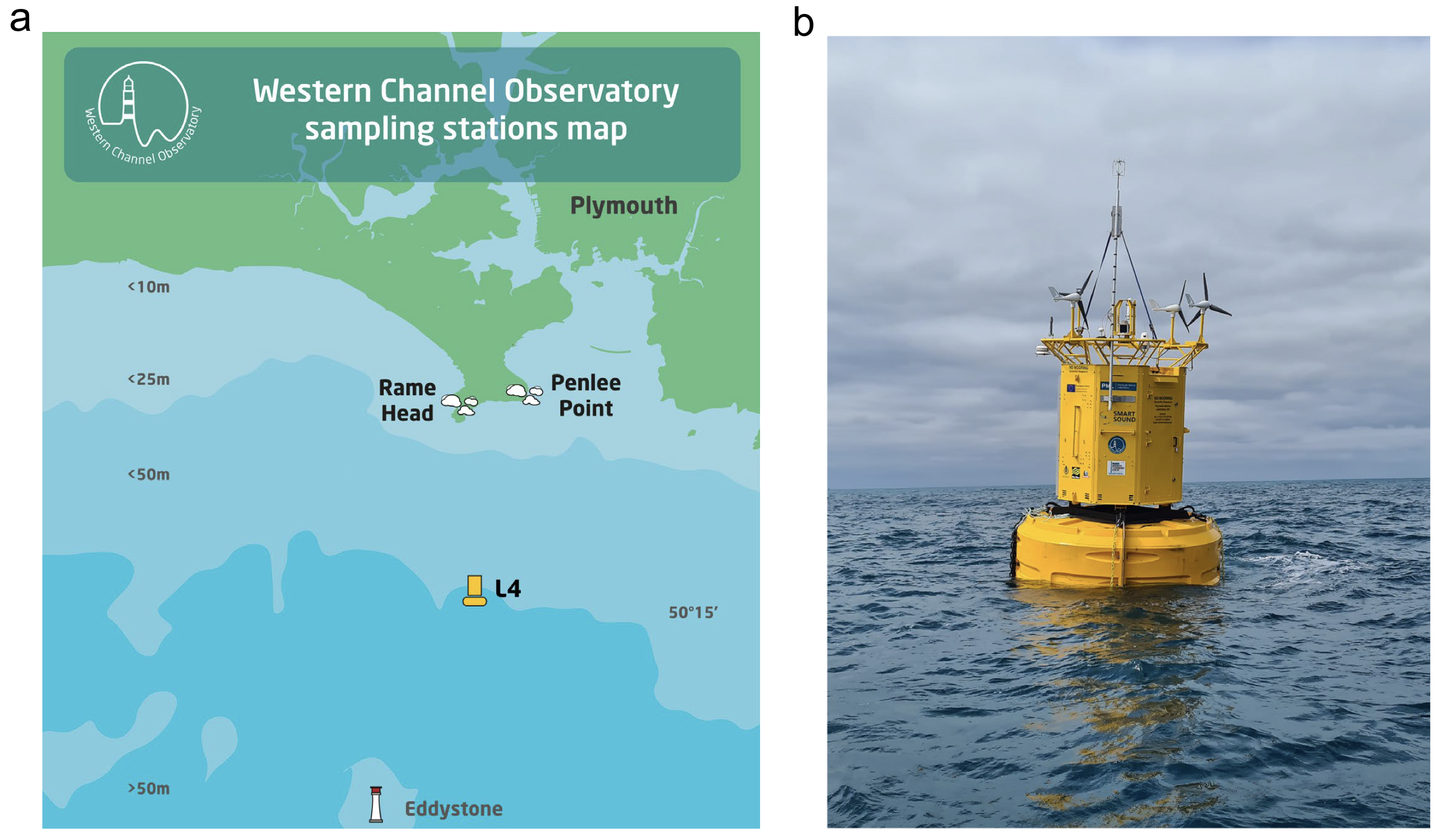


**Figure S1** **Collection of seawater samples at L4.** **a** Location of the station L4 (50^◦^15.00′ N, 4^◦^13.02′ W) in the Western English Channel, UK. **b** The data buoy used.


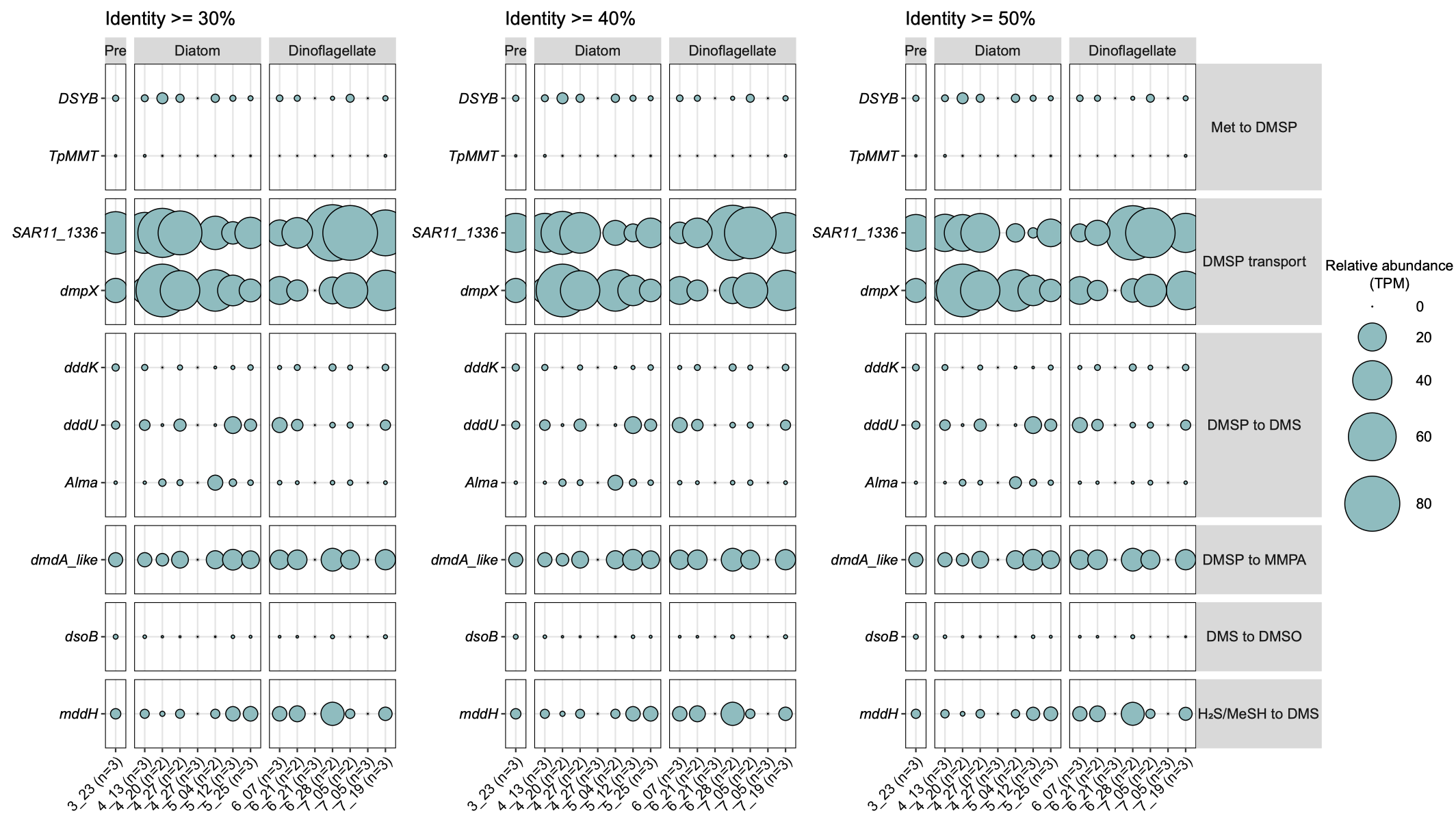


**Figure S2** **Sensitivity of transcript abundance patterns to sequence identity thresholds used for DIAMOND BLASTP annotation against ratified sequences.** Genes were identified by DIAMOND BLASTP with a more permissive identity threshold (30%), the threshold used in the main analysis (40%), and a more stringent threshold (50%).


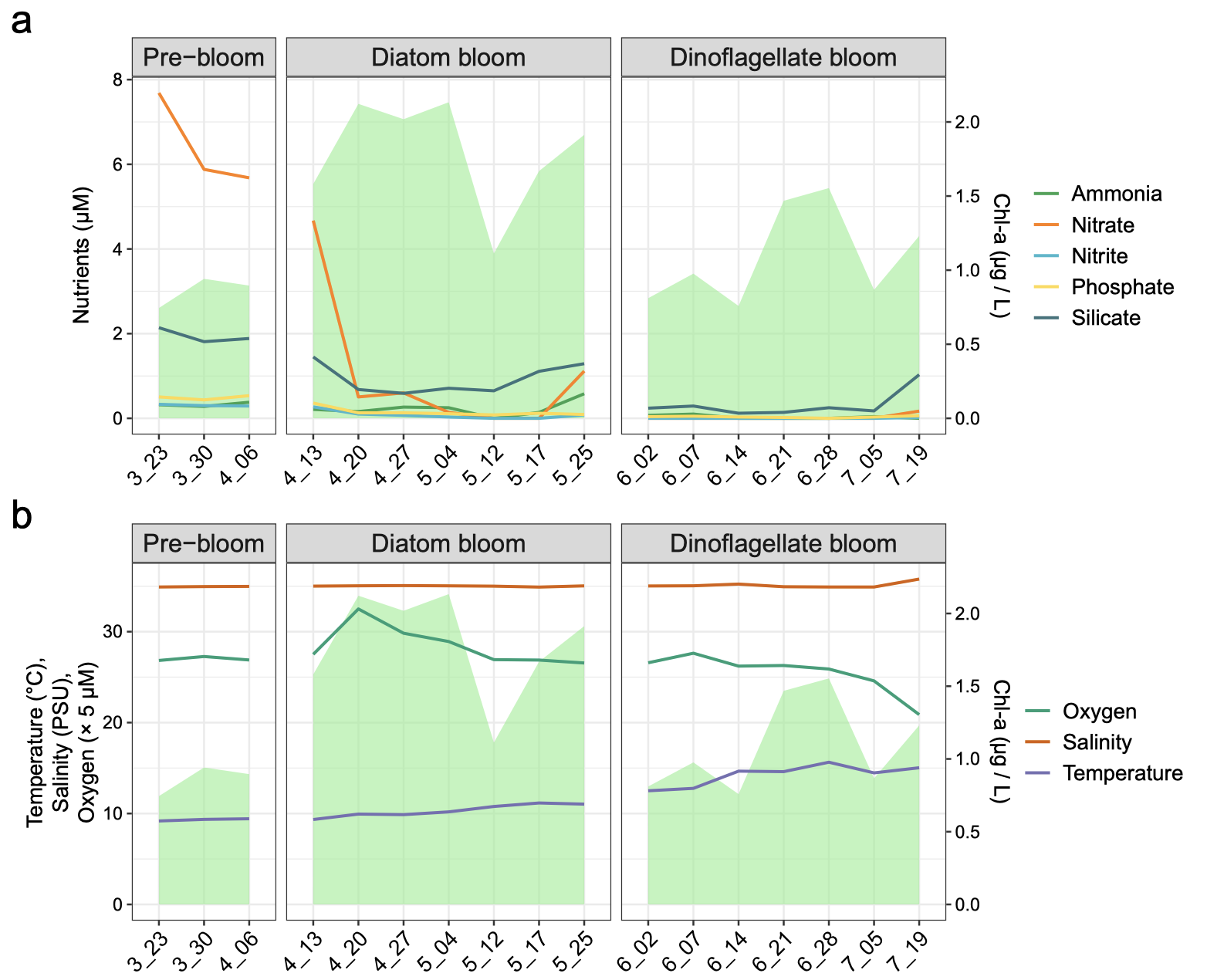


**Figure S3** **Environmental parameters at L4 from 23 March to 19 July 2021.** **a** Nitrite, nitrate, ammonia, silicate, and phosphate concentrations. **b** Temperature, salinity and oxygen concentrations. Chl-a concentrations were plotted as the light green areas in the background.


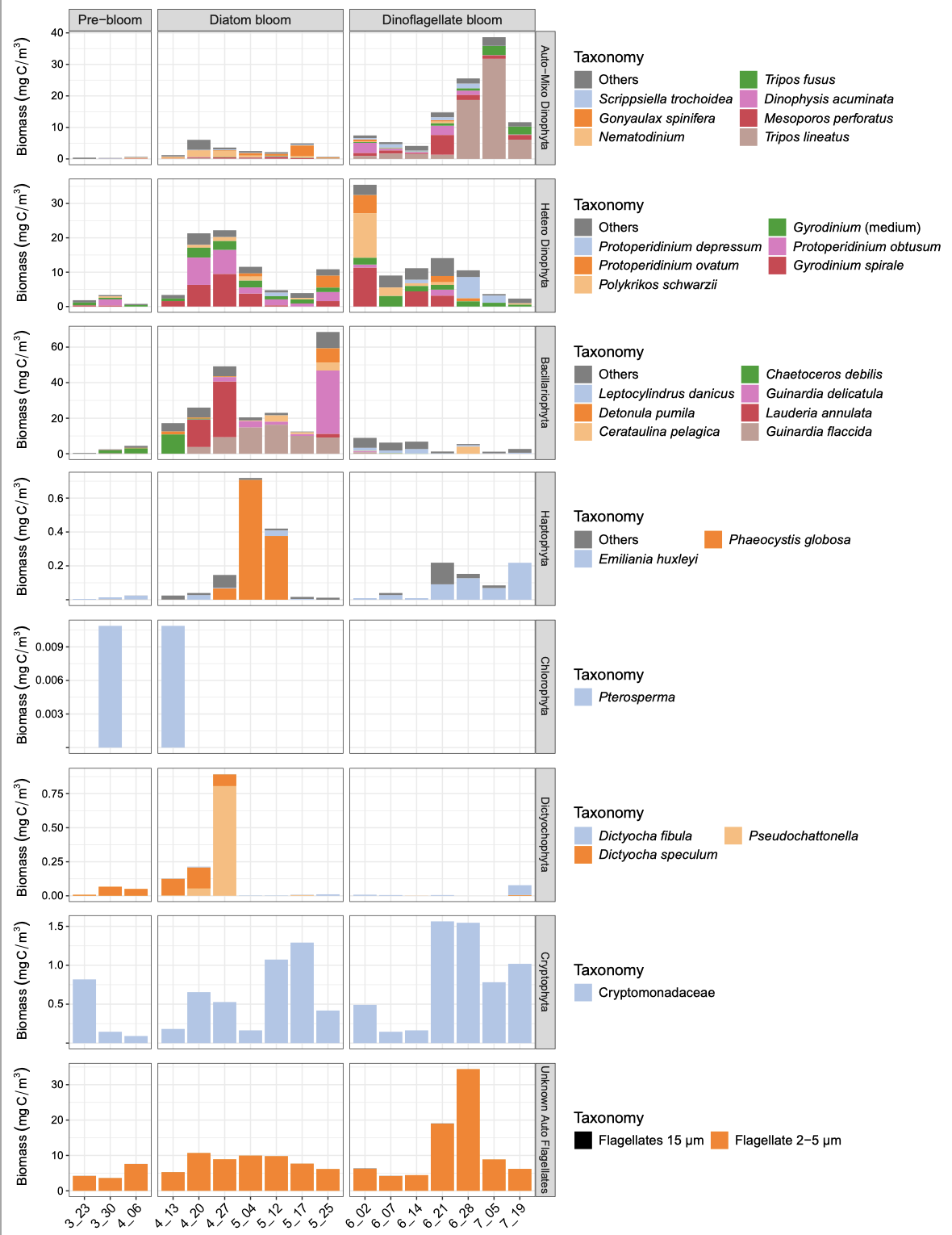


**Figure S4** **Community composition of dominant microalgae groups (shown in Fig. 2) detected in L4 waters from 23 March to 19 July 2021.**


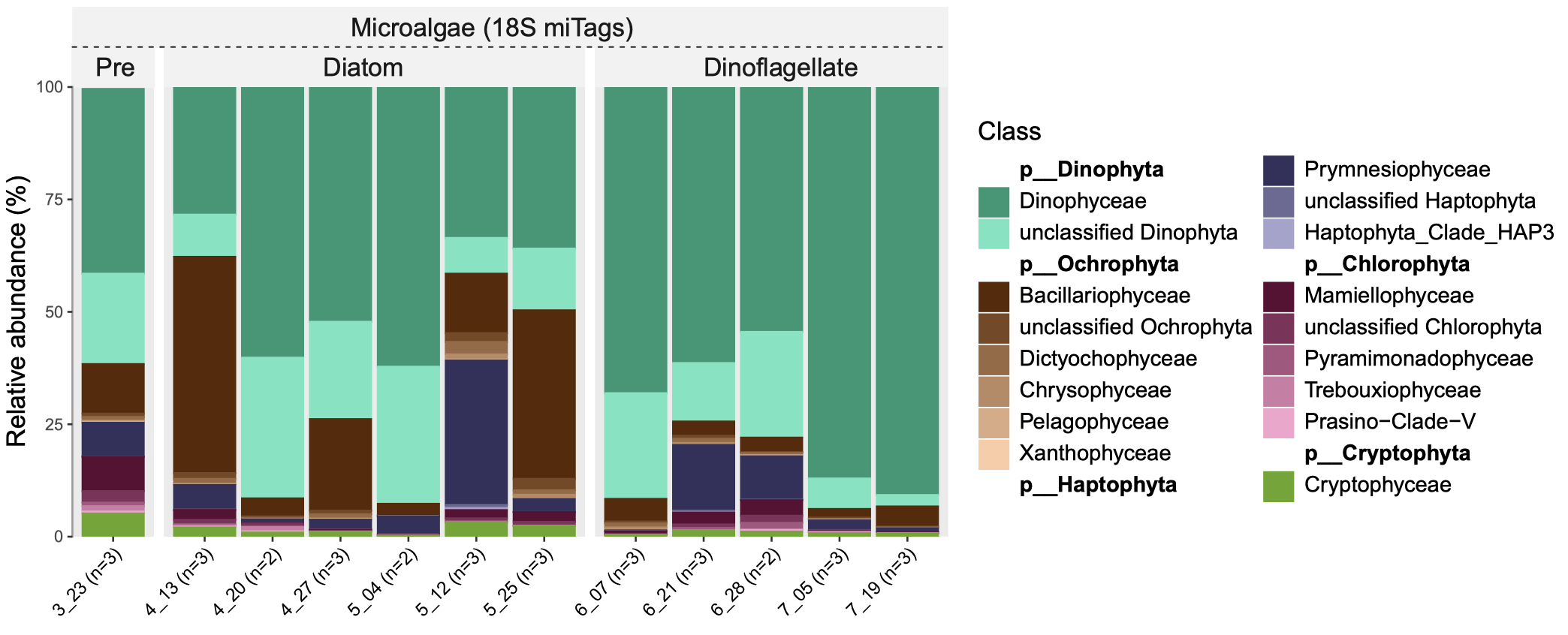


**Figure S5** **Microalgae community composition at L4 from 23 March to 19 July 2021, revealed by metagenome derived 18S rRNA gene reads (18S miTags).** The dominant classes are shown and grouped at phylum level. Pre, pre-bloom; Diatom, diatom bloom; Dinoflagellate, dinoflagellate bloom.


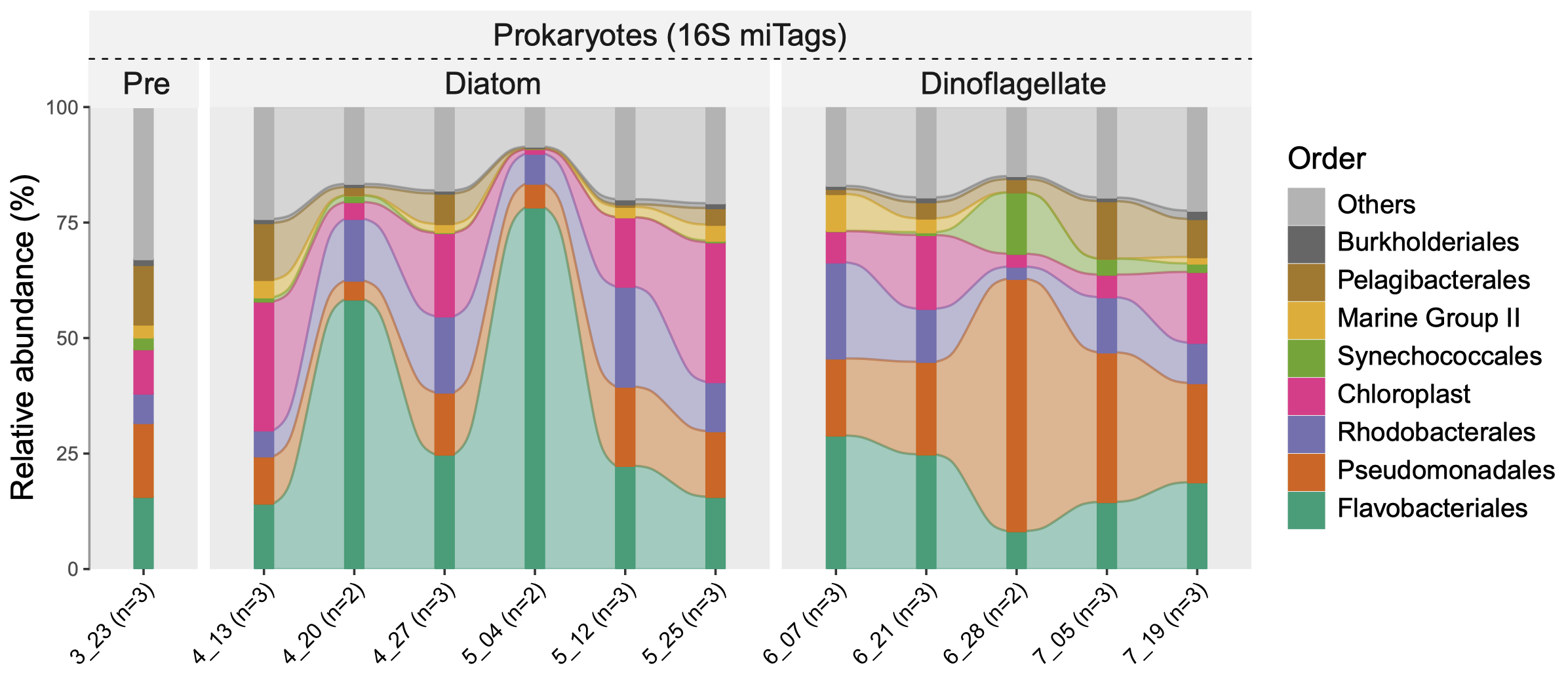


**Figure S6** **Prokaryotic community at L4 from 23 March to 19 July 2021, revealed by metagenome derived 16S rRNA gene reads (16S miTags).** The dominant orders are shown. Pre, pre-bloom; Diatom, diatom bloom; Dinoflagellate, dinoflagellate bloom.

**
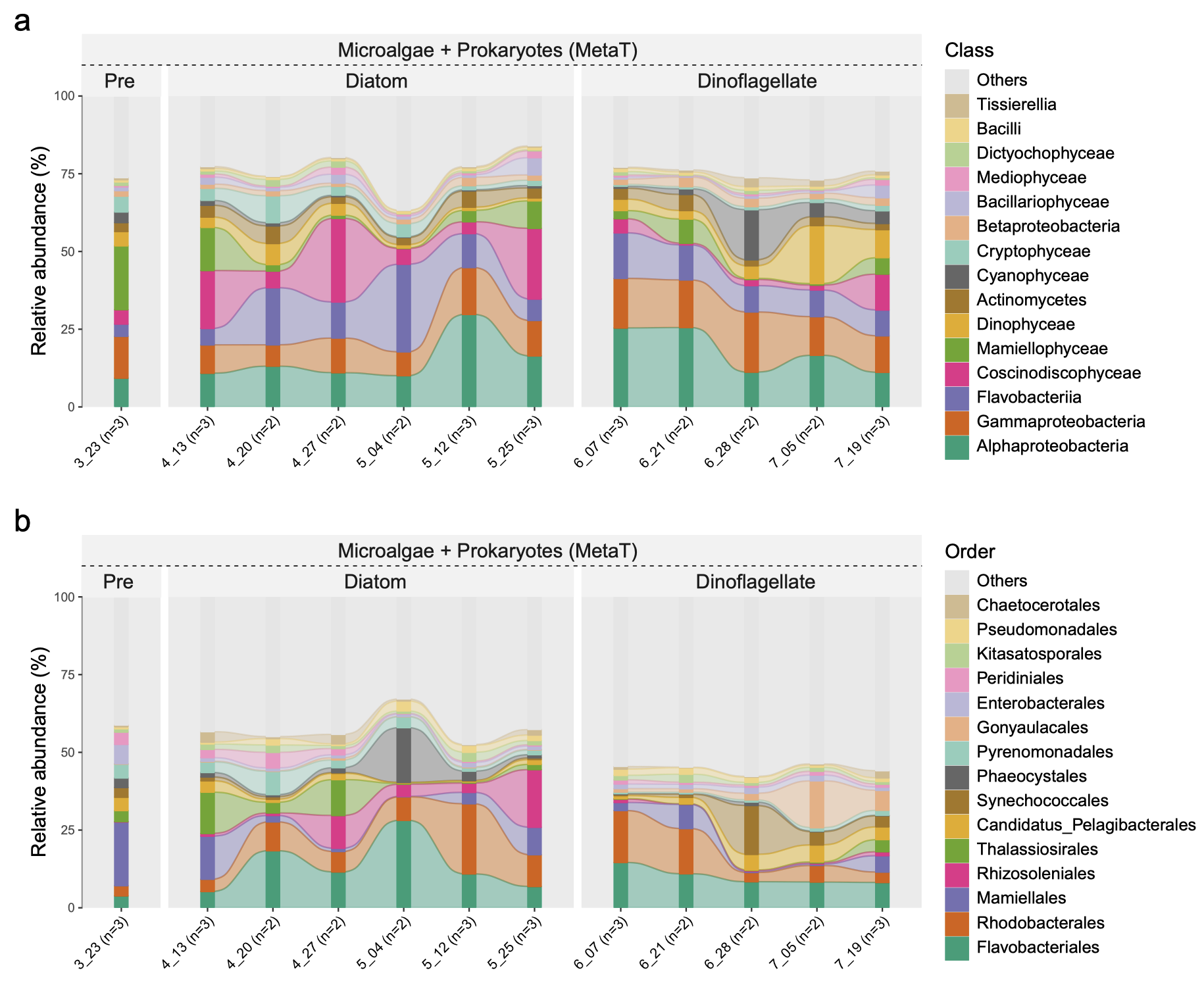
**

**Figure S7** **Plankton (microalgae and prokaryotes) community at L4 from 23 March to 19 July 2021 based on metatranscriptomic reads.** The dominant classes (**a**) and orders (**b**) are shown. MetaT, metatranscriptome. Pre, pre-bloom; Diatom, diatom bloom; Dinoflagellate, dinoflagellate bloom.


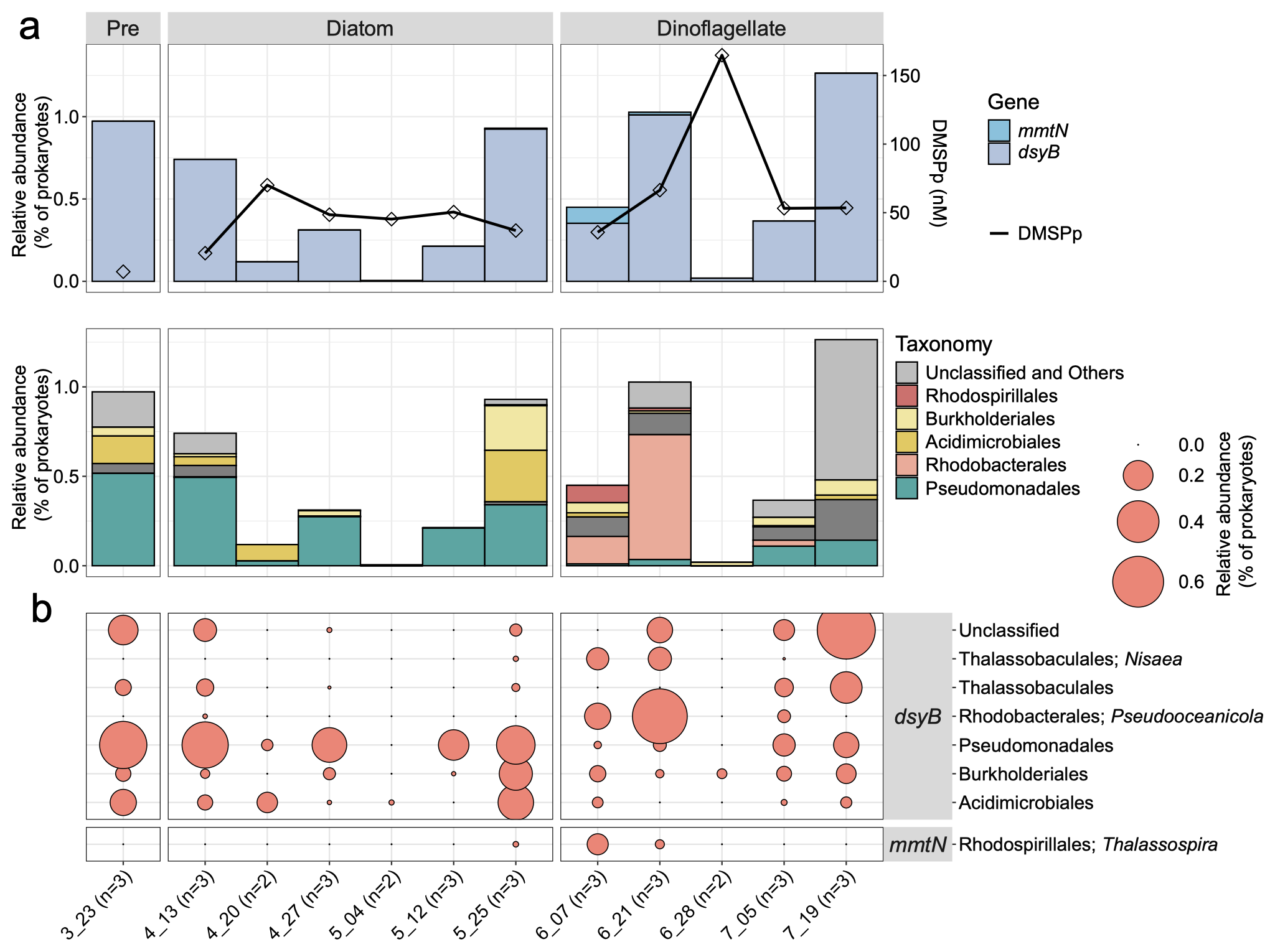


**Figure S8 Relative** **gene abundance of known bacterial DMSP synthesis genes at L4 from 23 March to 19 July 2021. a** Relative abundance and order-level taxonomic profiles of all detected DMSP synthase genes. DMSPp concentrations in corresponding samples are shown. **b** Taxonomic composition of each DMSP synthase gene. Biological replicate counts (n) are shown in parentheses after each sample name. Pre, pre-bloom; Diatom, diatom bloom; Dinoflagellate, dinoflagellate bloom.

**
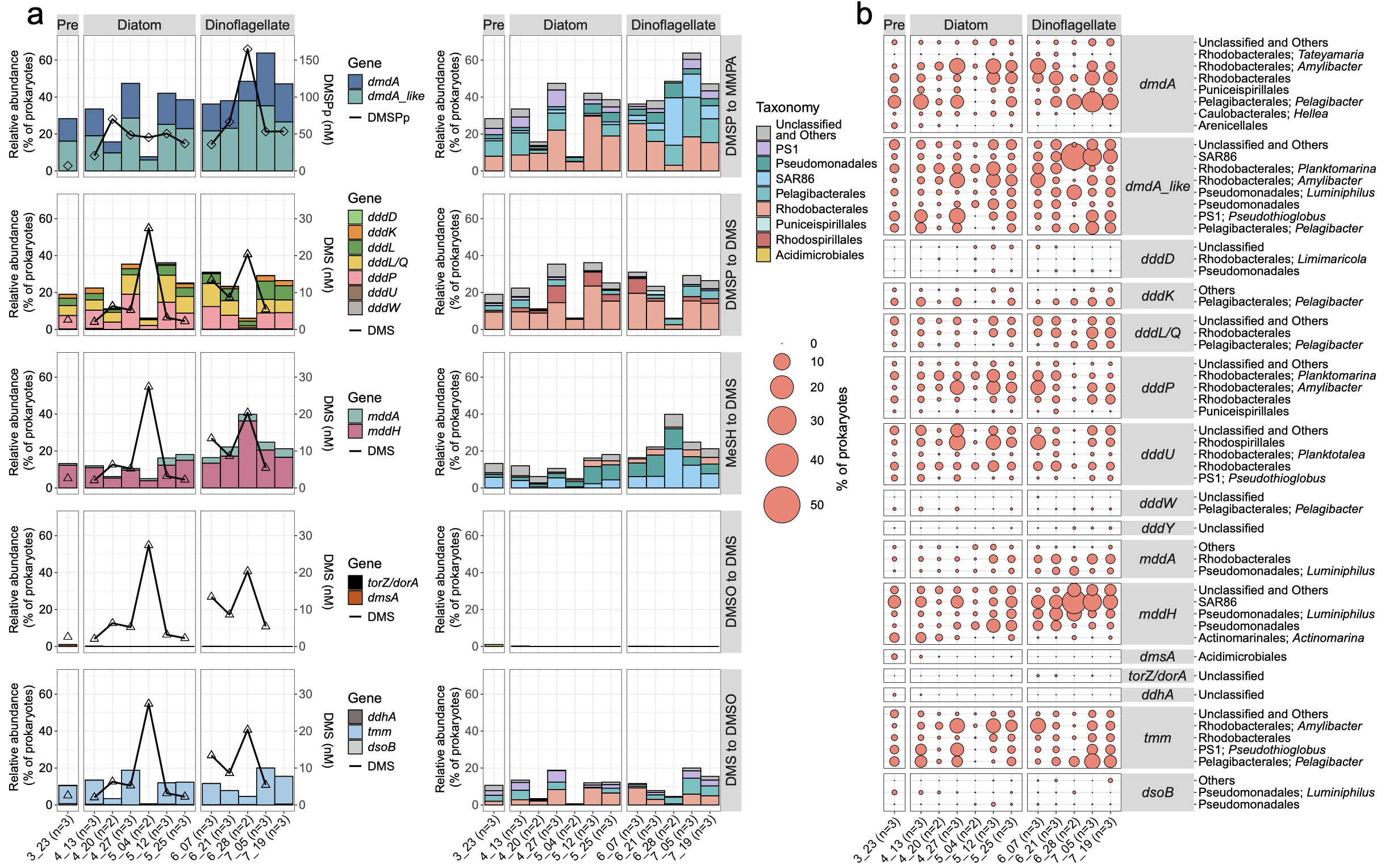
**

**Figure S9 Relative abundance of genes responsible for DMSP demethylation, DMSP/H_2_S/MeSH/DMSO-dependent DMS production, and DMS degradation at station L4 in the WEC from 23 March to 19 July 2021. a** Relative abundance and order-level taxonomic profiles of all detected genes. DMSPp or DMS concentrations in corresponding samples are shown. **b** Taxonomic composition of each gene. Biological replicate counts (n) are shown in parentheses after each sample name. Pre, pre-bloom; Diatom, diatom bloom; Dinoflagellate, dinoflagellate bloom.


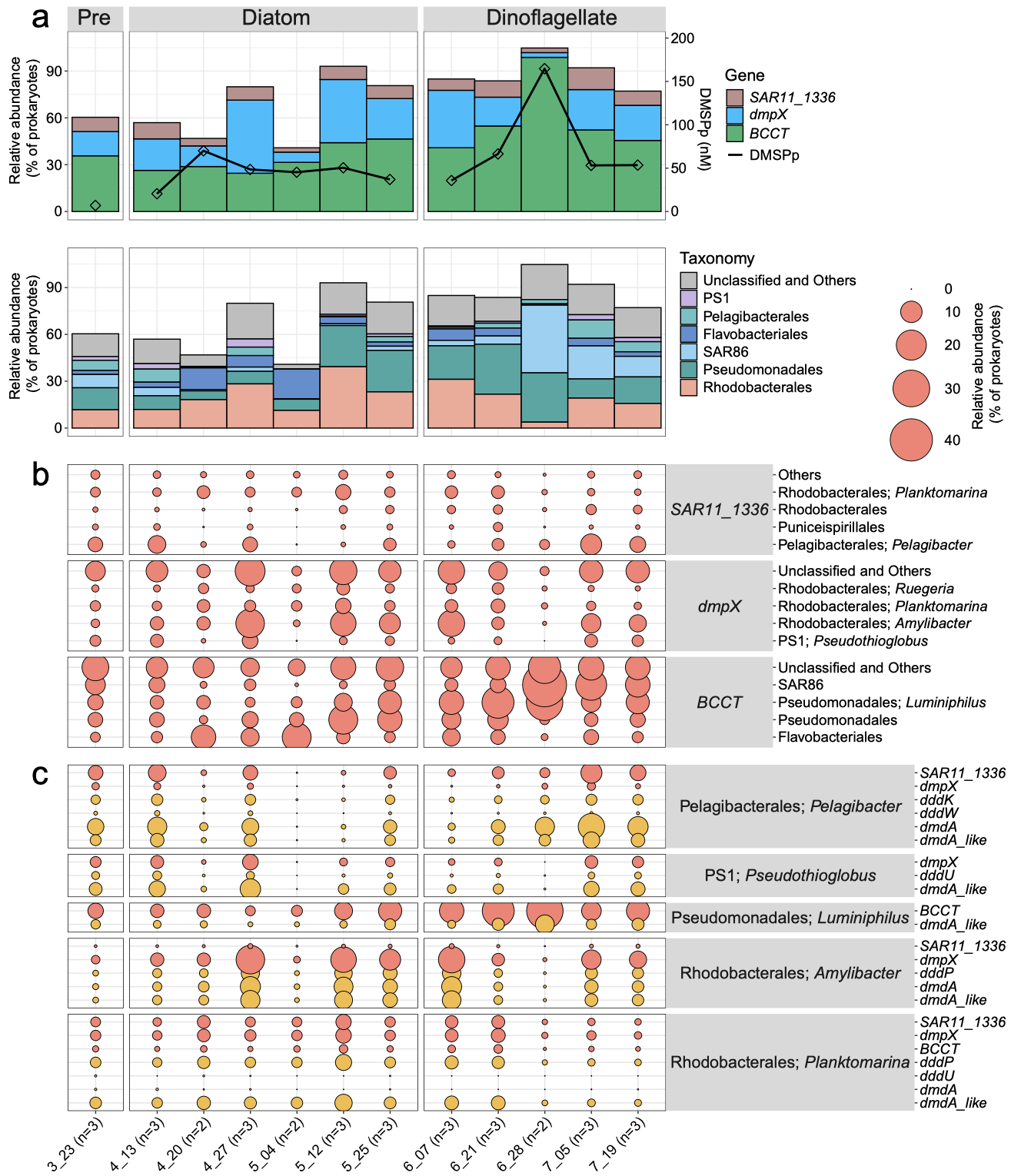


**Figure S10 Relative gene abundance of potential DMSP transporter genes at L4 from 23 March to 19 July 2021. a** Relative abundance and order-level taxonomic profiles of all detected DMSP transporter genes. DMSPp concentrations in corresponding samples are shown. **b** Taxonomic composition of each transporter gene. **c** Comparison of the relative abundance of DMSP transporter (red) and catabolic (yellow) genes from the five predicted DMSP degraders at L4. Biological replicate counts (n) are shown in parentheses after each sample name. Pre, pre-bloom; Diatom, diatom bloom; Dinoflagellate, dinoflagellate bloom.


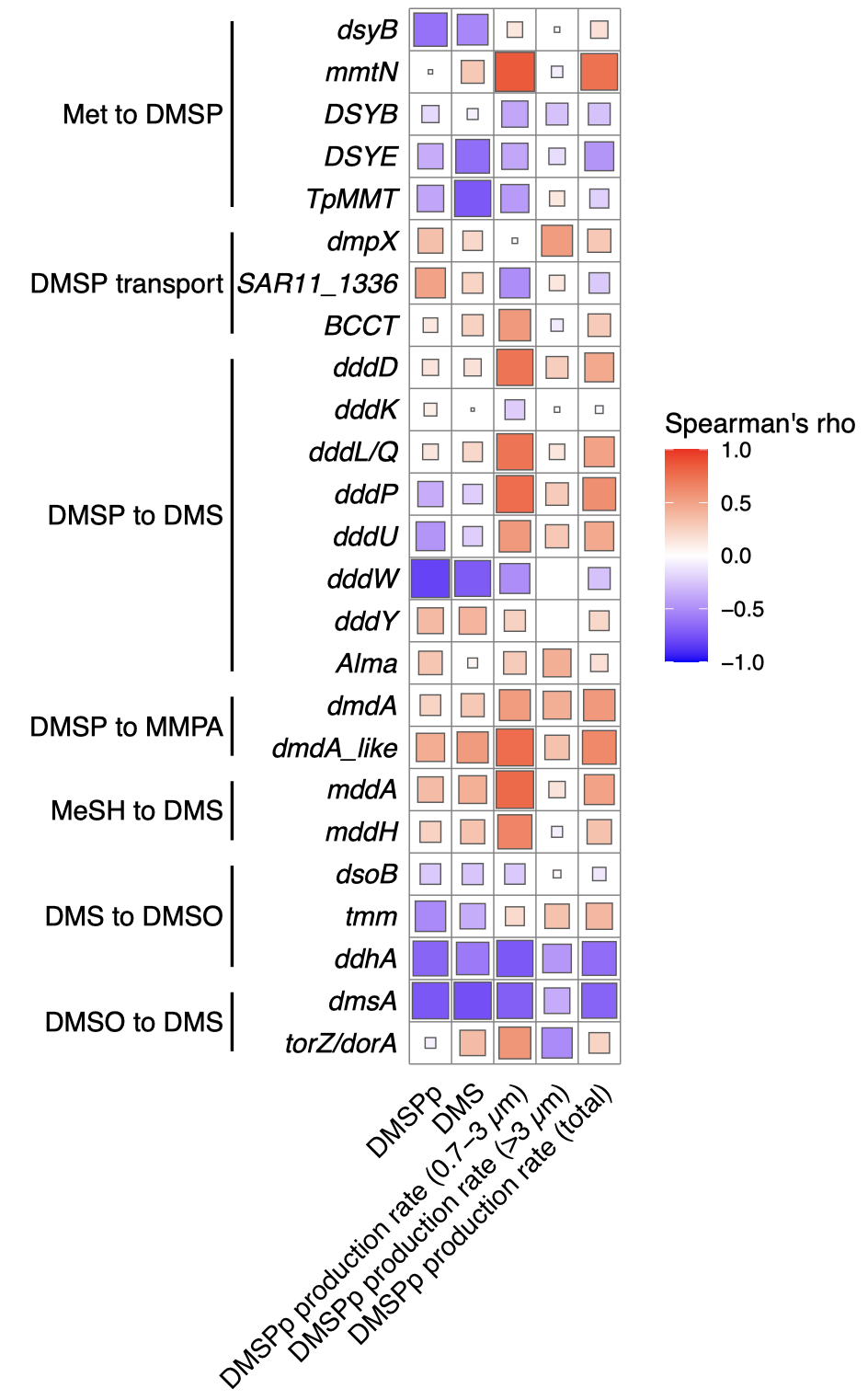


**Figure S11 Spearman correlation analyses between DMSPp concentrations, DMS concentrations, and DMSPp production rates, and the transcript abundance of genes involved in DMSP synthesis, transport, and catabolism, as well as DMS production and consumption.** Spearman’s rho values are shown only for significant correlations (**P* < 0.05, ***P* < 0.01, ****P* < 0.001). No statistically significant correlations were observed in this analysis; therefore, no significance asterisks appear in the figure.


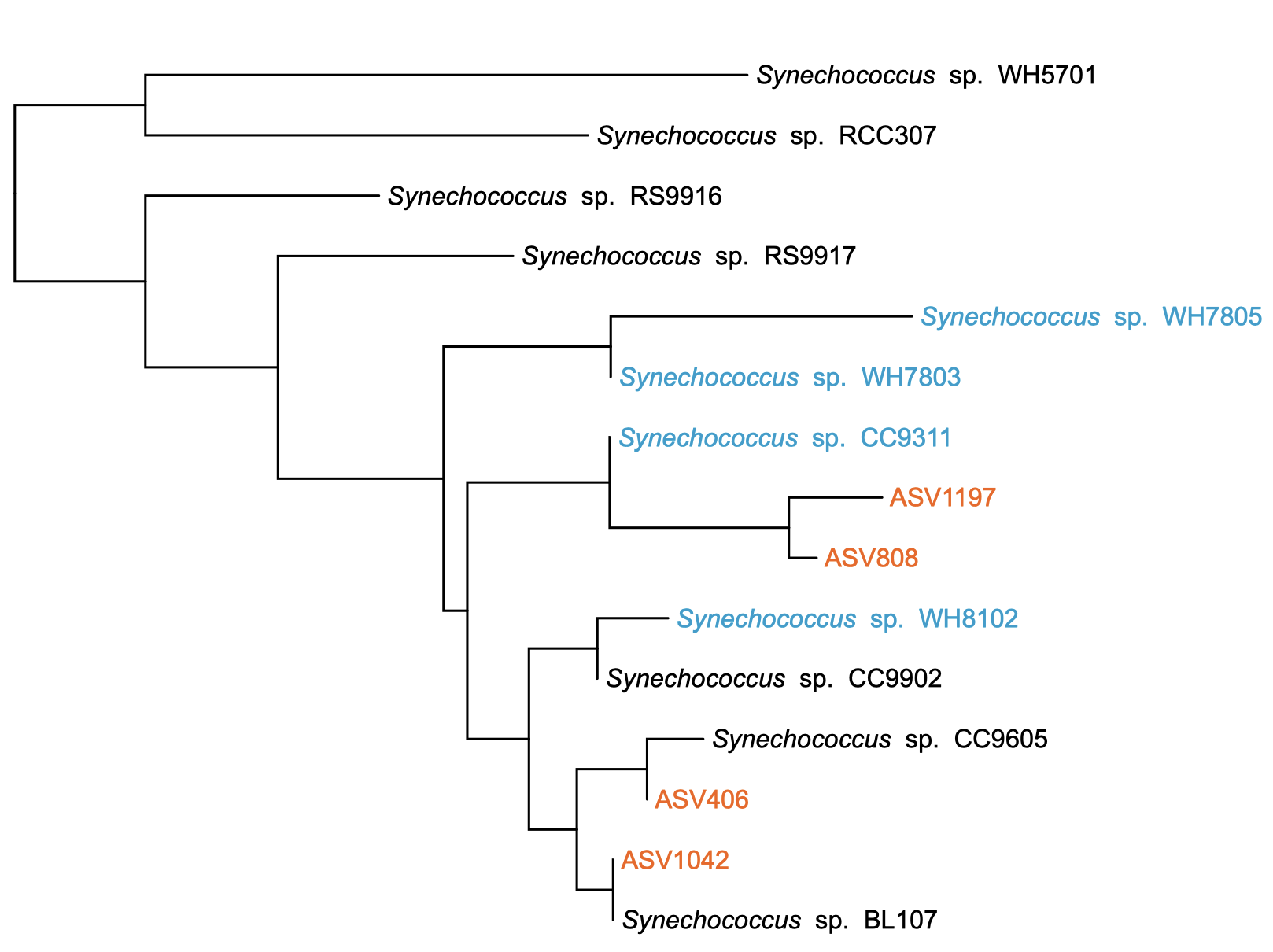


**Figure S12 Neighbor-joining (NJ) phylogeny of 16S rRNA genes from *Synechococcus* strains.** *Synechococcus* ASVs identified from L4 by amplicon sequencing are in orange. Axenic *Synechococcus* strains used for testing DMSP production are in cyan, none of which accumulated DMSP above the detection limit of the gas chromatography (GC) instrument. Other sequences were retrieved from NCBI.


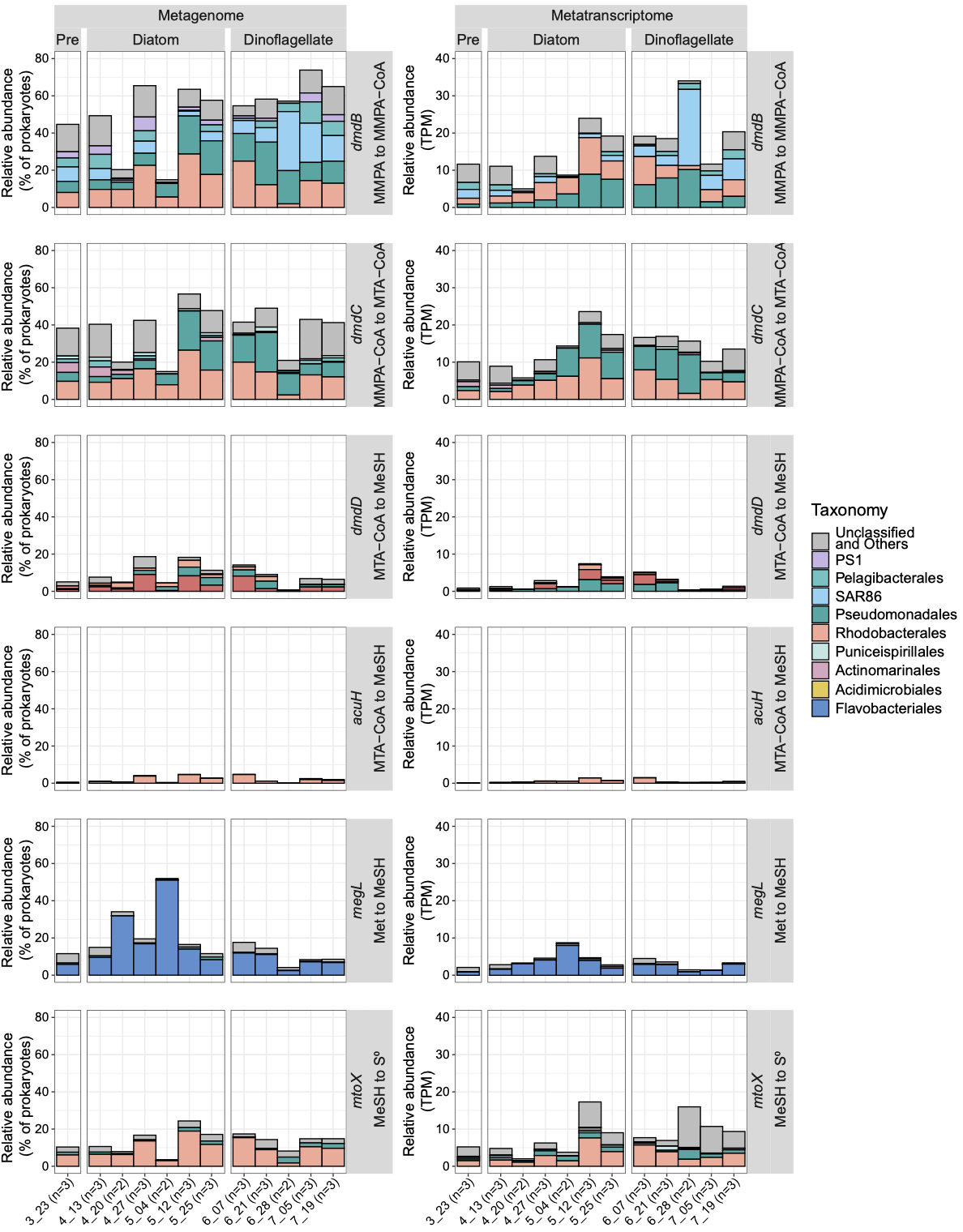


**Figure S13 Relative abundance of genes (left) and transcripts (right) related to MeSH production and degradation at L4 from 23 March to 19 July 2021.** Genes involved in DMSP-dependent MeSH production (*dmdB*, *dmdC*, *dmdD*, and *acuH*), Met-dependent MeSH production gene (*megL*), and MeSH degradation (*mtoX*) are shown. Note, *dmdD* and *acuH* are the key genes for MeSH generation. Order-level taxonomic composition of each gene is shown. Biological replicate counts (n) are shown in parentheses after each sample name. Pre, pre-bloom; Diatom, diatom bloom; Dinoflagellate, dinoflagellate bloom; TPM, transcripts per million reads.


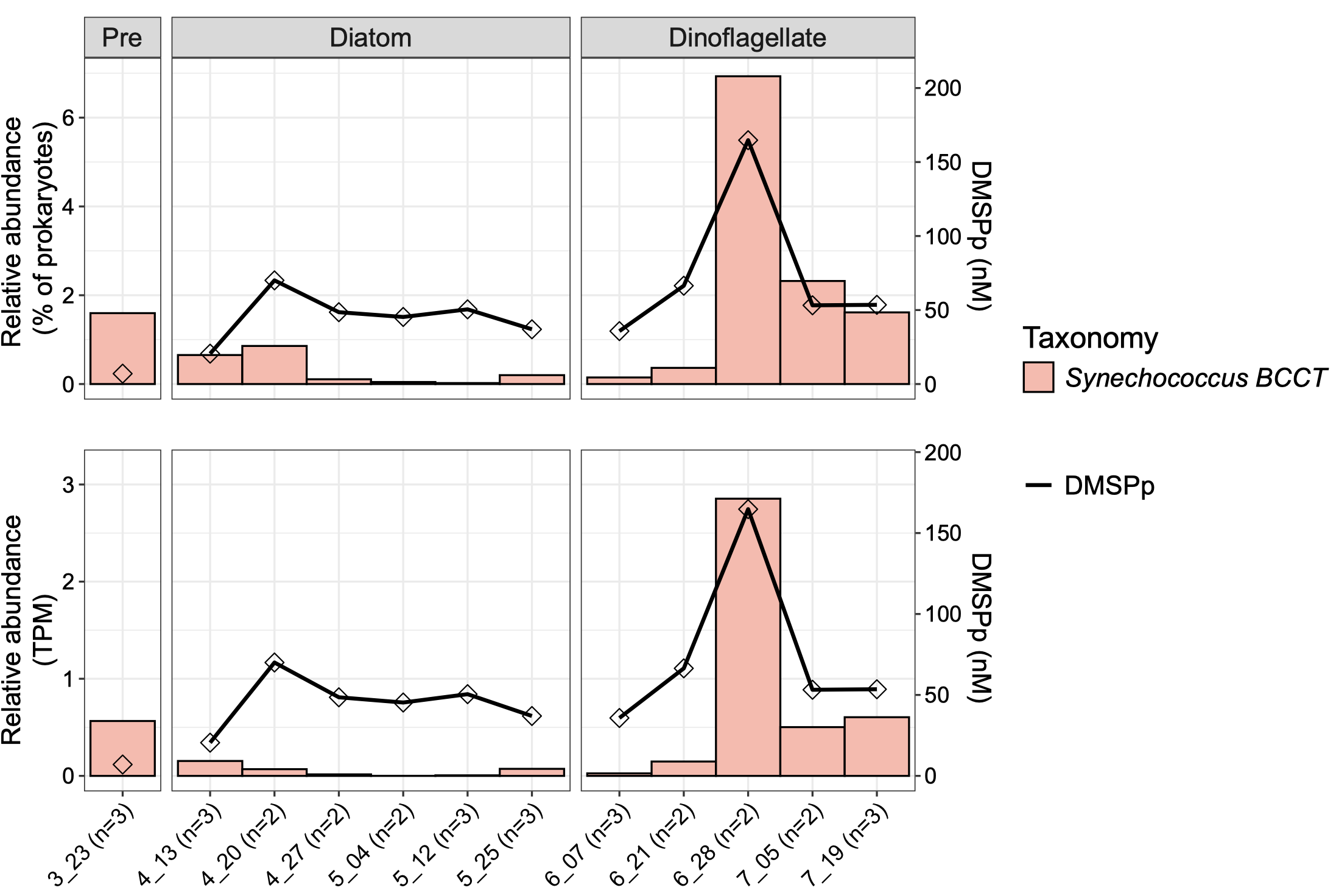


**Figure S14 Relative abundance of *Synechococcus* *BCCT* genes (top) and transcripts (bottom) at L4 from 23 March to 19 July 2021.** DMSPp concentrations in corresponding samples are shown. Biological replicate counts (n) are shown in parentheses after each sample name. Pre, pre-bloom; Diatom, diatom bloom; Dinoflagellate, dinoflagellate bloom; TPM, transcripts per million reads.
